# Synthetic Biology Approaches to Hydrocarbon Biosensors: A Review

**DOI:** 10.3389/fbioe.2021.804234

**Published:** 2022-01-10

**Authors:** Claudia F. Moratti, Colin Scott, Nicholas V. Coleman

**Affiliations:** ^1^ School of Life and Environmental Science, Faculty of Science, University of Sydney, Sydney, NSW, Australia; ^2^ CSIRO Synthetic Biology Future Science Platform, Canberra, ACT, Australia

**Keywords:** biosensor, hydrocarbon, alkane, alkene, monooxygenase, transcription factor, regulation, bacteria

## Abstract

Monooxygenases are a class of enzymes that facilitate the bacterial degradation of alkanes and alkenes. The regulatory components associated with monooxygenases are nature’s own hydrocarbon sensors, and once functionally characterised, these components can be used to create rapid, inexpensive and sensitive biosensors for use in applications such as bioremediation and metabolic engineering. Many bacterial monooxygenases have been identified, yet the regulation of only a few of these have been investigated in detail. A wealth of genetic and functional diversity of regulatory enzymes and promoter elements still remains unexplored and unexploited, both in published genome sequences and in yet-to-be-cultured bacteria. In this review we examine in detail the current state of research on monooxygenase gene regulation, and on the development of transcription-factor-based microbial biosensors for detection of alkanes and alkenes. A new framework for the systematic characterisation of the underlying genetic components and for further development of biosensors is presented, and we identify focus areas that should be targeted to enable progression of more biosensor candidates to commercialisation and deployment in industry and in the environment.

## 1 Introduction

Synthetic biology repurposes the native functions of organisms to engineer creative solutions to problems. The metabolic diversity of bacteria is among their most useful properties for synthetic biology; this feature is the product of millions of years of evolutionary adaptation to diverse niches, nutrients, and stresses. Hydrocarbon-metabolising bacteria are especially interesting for synthetic biology applications, including bioremediation (Das and Chandran, 2011), biocatalysis (Coscolín et al., 2019), and biosensors (Sticher et al., 1997).

Nearly eighty different bacterial genera have been identified as degraders of at least one petrochemical, although only a fraction are genetically or biochemically well-characterised. Monooxygenases are the key enzymes responsible for bacterial degradation of alkanes and alkenes in such bacteria, and the regulatory systems of monooxygenases are therefore of significant interest. There is a need to better characterise these sensing systems to better understand the metabolic diversity of bacteria and to capitalise on these biological switches.

Several eukaryotic species are also capable of hydrocarbon degradation, including many genera of fungi and yeast, and one alga (Prince, 2010). Similarly to bacterial systems, monooxygenases are responsible for the eukaryotic metabolism of aliphatic alkanes. To date only the cytochrome P450 class of monooxygenases have been identified to perform this role (Das and Chandran, 2011; Prenafeta-Boldu et al., 2019) and only under aerobic conditions, unlike in bacteria where hydrocarbon metabolism is facilitated by several classes of monooxygenases and can also occur anaerobically. In fungal systems, other types of hydrocarbons including alkenes and polycyclic aromatics are typically only partially degraded and it is rare for hydrocarbons to act as a sole carbon source (Prince, 2010; Prenafeta-Boldu et al., 2019). Eukaryotic hydrocarbon degradation has already been extensively reviewed (Cerniglia and Crow, 1981; Prince, 2010; Das and Chandran, 2011; Beier et al., 2014; Prenafeta-Boldu et al., 2019) and so is not included in the scope of this review. Moreover, the variety of monooxygenases involved in bacterial hydrocarbon degradation is interesting and warrants a narrow focus, especially when considering these systems for biotechnology applications.

**FIGURE 1:**
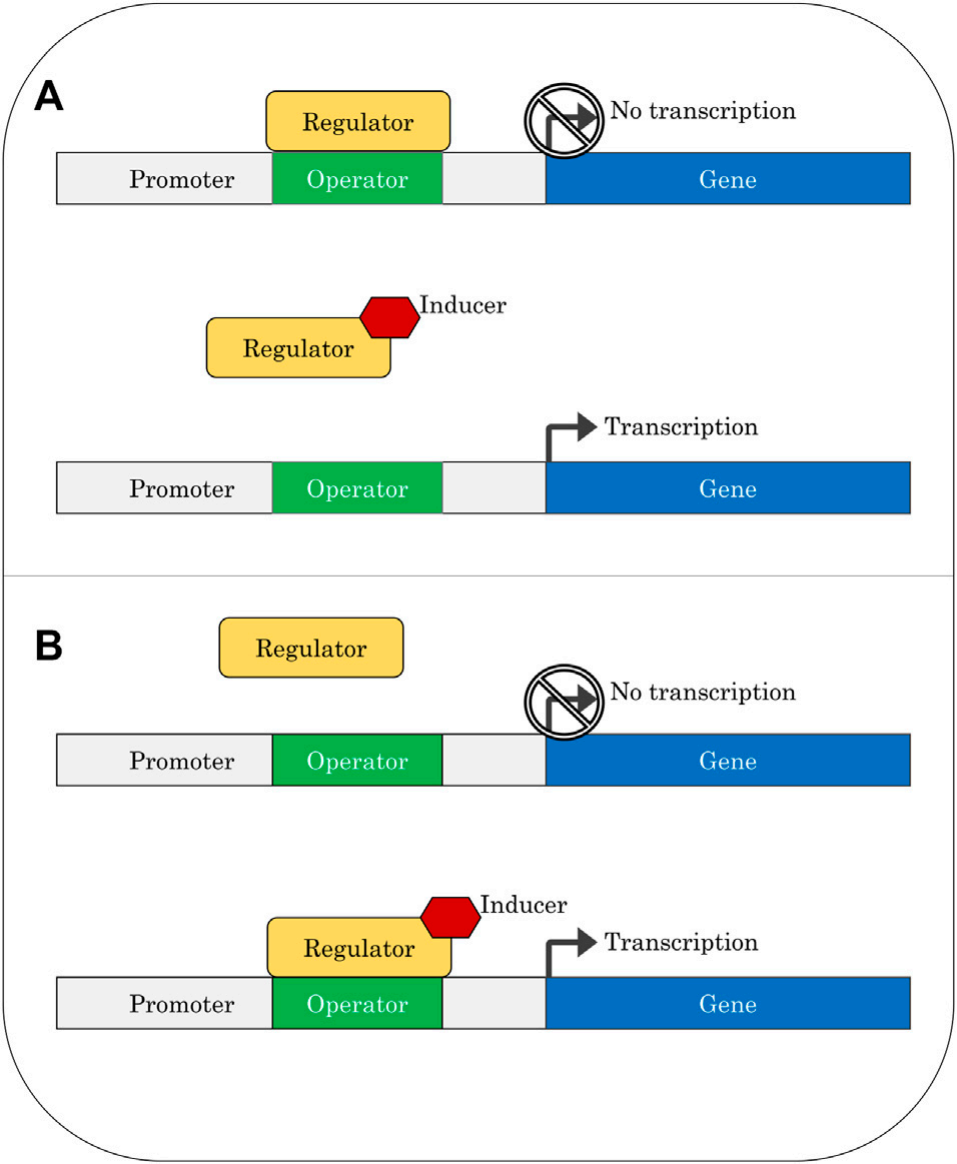
Gene regulation *via* repressor protein **(A)** or activator protein **(B)**.

Hydrocarbon-sensing systems consist of regulatory proteins (transcription factors) that bind to an inducer and then interact with operator sequences near the promoter of the gene being controlled, resulting in a change in expression levels of that gene (Figure 1). In some cases, the sensing system is divided into two proteins, one which binds the inducer, and another which interacts with the operator; the induction signal in these cases is passed from the first protein to the second. A promoter is a sequence of DNA upstream of a gene that recruits RNA polymerase for transcription (Browning and Busby, 2016). Operators act as binding sites for specific transcription factors and have features including direct or inverted repeats. An inducer is a compound that interacts with the regulatory protein in a way which changes the binding of the protein to the operator.

A regulatory protein is considered a repressor if it binds to the operator in the absence of an inducer, preventing transcription. An inducer will relieve repression by binding to the protein in a way that prevents the protein from remaining bound to the operator. In contrast, activators are regulatory proteins that only bind to the operator once they have complexed with an inducer; in these cases, transcription will be switched on after binding of the activator protein. Transcription-factor-based biosensors can be easily constructed from these regulatory components by replacing the metabolic genes normally controlled by the system with a readily detectable output such as fluorescence. The broad use of bacterial two-component systems in biosensors has been recently reviewed (Lazar and Tabor, 2021).

There has been much research into the bacterial metabolism and detection of aromatic hydrocarbons (Chauhan et al., 2008; Plotnikova et al., 2016; Reineke et al., 2020). Less is known about metabolism and sensing of aliphatic hydrocarbons, in particular the shorter chain gaseous compounds. Development of biosensors for detection of *n*-alkanes and *n*-alkenes has mostly been aimed at monitoring the clean-up of oil spills in seawater (Harayama et al., 2004; Tecon and Van der Meer, 2008; Tecon et al., 2010; Das and Chandran, 2011; Reed et al., 2012; Zhang et al., 2012a; Sevilla et al., 2017; Varjani, 2017), with a consequent focus on biosensor systems detecting octane (Jiang et al., 2020) and bacterial hosts suited to marine environments (Sevilla et al., 2015). The limited scope of research on aliphatic biosensors is unfortunate given their potentially many applications.

Aliphatic hydrocarbon biosensors are potentially useful in the chemical industry for real-time monitoring of reactants or products (Minak-Bernero et al., 2004), in the fresh produce industry for the detection of ethene (Janssen et al., 2014), and in the natural gas industry for both safety and maintenance applications (e.g., leak detection) (Nandimandalam et al., 2018). There has also been interest in the biofuels industry in biosensors for detecting the intracellular concentrations of biosynthesized alkanes (Wu et al., 2015). Finally, there are potential medical applications for alkane biosensors in analysis of breath samples for lung cancer diagnosis (Tan et al., 2016).

This review will provide a framework for the development of transcription-factor based biosensors to help guide future development efforts. The framework will then be used to assess the current research landscape in the case of aliphatic hydrocarbons, including review of the bacteria and catabolic enzymes associated with these substrates, the types of regulatory genes involved, and consideration of the limiting factors in the research development pipeline. Methods commonly used to fulfil the requirements of each step of the framework will also be summarised throughout.

## 2 A Framework for Bacterial Biosensor Development

A framework has been developed here to show the key stages in the development of transcription-factor-based biosensors; this includes clear research goals that must be achieved to progress from concept to deployment. When applied to the biosensor literature to date, this framework allows identification of the limiting steps, which is important to focus funding and research in the areas which will provide the most reward for effort.

The first step in the development of any biosensor is the identification of a biological part that could be repurposed for a sensing application; this might be an enzyme, antibody or nucleic acid (van der Meer, 2010). Research then progresses to characterisation, where the function of the candidate sensor is confirmed. This is usually done *via* the deletion and/or complementation of the gene and/or promoter element in the presence of the predicted inducer compound(s). Characterisation may be done in stages depending on how the sensing system was first discovered. For example, if it was found associated with specific catabolic genes, the function of these is often first confirmed before the associated sensing elements are characterised.

The third checkpoint in biosensor development is proof-of-concept in a controlled laboratory environment. At this stage, various key parameters such as sensitivity, selectivity, shelf life and response time would be determined. Next is the development phase, where the biosensor circuitry and context are adapted with the intention of optimising those key parameters. Finally, the device can proceed to be engineered for market deployment. This stage includes activities such as: seeking regulatory approval, benchmarking analysis, production scale-up, process optimisation, cellular immobilisation, and company formation.

Applying the above framework to the specific case of aliphatic hydrocarbon biosensors typically involves two rounds of identification and characterisation. First, a monooxygenase-encoding gene is identified, and the corresponding enzyme function is characterised. Secondly, the transcription factor responsible for monooxygenase expression is identified and the regulatory mechanism of the system is characterised. There will be a strong focus on the characterisation strategies for these regulatory mechanisms in this review because it is at this stage where greater research efforts are most needed, as will be made apparent.

## 3 Identification and Characterisation of Monooxygenases

Identification of a biological part may be deliberate or coincidental, rigorous or speculative. Bioinformatic detection could be targeted at the transcription factor itself, or at the associated catabolic genes. The identification stage typically involves the study of pure cultures of bacteria, database mining and/or metagenomics.

The identification of the monooxygenase responsible for aliphatic hydrocarbon oxidation is a natural first step in biosensor development for this class of compounds. The rationale for this is two-fold. Firstly, monooxygenase genes make better targets for bioprospecting (Holmes and Coleman, 2008) than the associated regulatory genes, which tend to be more diverse and thus more difficult to detect by PCR or sequence analysis. Secondly, in many cases, the inducers of aliphatic hydrocarbon degradation genes are not the alkanes or alkenes, but rather the downstream metabolites (alcohols, aldehydes, epoxides) (Kurth et al., 2008) and it is the monooxygenase enzymes that play the crucial role of converting the hydrocarbon of interest into the inducer molecule.

Monooxygenases are responsible for the oxidation of methyl or methylene groups in n-alkanes, which is the first step in the catabolism of n-alkanes in all aerobic bacteria. The alcohols produced by this catalysis can then be oxidised into aldehydes or ketones, and then ultimately into fatty acids (Van Beilen et al., 2003; Kotani et al., 2006). Monooxygenases are of equal importance for alkene oxidation, with the resultant epoxides processed *via* a series of coenzyme M or glutathione-mediated reactions (Vlieg et al., 2000; Ensign, 2001; Mattes et al., 2010). It is not uncommon for one bacterial isolate to possess multiple monooxygenases of different types (Rojo, 2009), which may include both alkane and alkene-oxidising enzymes.

### 3.1 Methods for Identifying the Presence of Monooxygenases in Bacterial Species

Monooxygenase genes are most commonly identified in cultures obtained from enrichment and isolation on aliphatic hydrocarbons as the sole carbon source (Holmes and Coleman, 2008). Alternatively, these genes can be retrieved by metagenomic analysis, preferably using DNA from environments enriched in hydrocarbons (Musumeci et al., 2017; Gacesa et al., 2018), or by trawling pre-existing sequence data, which is now abundant due to the decreased costs of DNA sequencing. Monooxygenase homologues fall into distinct classes (Table 1), with known conserved sequence regions, and thus they can easily be identified purely based on sequence analysis in genomes or metagenomes, and functional approaches are not required at the gene discovery stage. The main classes of monooxygenases and their relevant properties are summarised in Table 1.

**TABLE 1:** Organisation and properties of bacterial monooxygenases involved in aliphatic hydrocarbon degradation.

Monooxygenase family		Description^a^	Substrate(s)	Inducer(s)	Example
**SDIMO:** Soluble di-iron monooxygenases	Group 1	Contains 4 protein subunits (α_2_β_2_γ_2_)-C-F-R, encoded by 6 genes Small and Ensign (1997), Zhou et al. (1999)	benzene, phenol, toluene, xylenes, styrene, naphthalene, indole, C_2_-C_6_ alkenes, C_2_ chloroalkenes, chloroform, isoprene Ensign et al. (1992)	benzene, toluene, xylenes, methylphenols, chlorophenols, C_2_-C_4_ (chloro)alkenes, epoxyisoprene Ensign. (1996), Arenghi et al. (1999), Arenghi et al. (2001), Silva-Jiménez et al. (2012), Crombie et al. (2015)	Propene monooxygenase XamoABCDEF from *Xanthobacter* Py2 Zhou et al. (1999)
	Group 3, includes **sMMOs**: soluble methane monooxygenases	Contains 4 protein subunits (α_2_β_2_γ_2_)-C-X-R encoded by 6 genes Murrell et al. (2000), Banerjee et al. (2019)	C_1_-C_9_ alkanes and haloalkanes, C_2_-C_4_ alkenes and haloalkenes, C_1_-C_2_ ethers, cyclohexane, benzene, toluene, styrene, pyridine, methanol Dubbels et al. (2007)	low Cu/biomass ratio (sMMO only), n-butanol (BMO) Hanson and Hanson (1996), Kurth et al. (2008)	Soluble methane monooxygenase MmoXYBZDC.from *Methylococcus capsulatus* Bath Stainthorpe et al. (1990), Rosenzweig et al. (1993)
	Group 4	Contains 3 protein subunits (αβ)-C-R encoded by 4 genes Saeki and Furuhashi (1994), Miuran and Dalton. (1995)	C_2_-C_10_ alkenes and haloalkenes, C_5_-C_9_ cycloalkenes Miuran and Dalton (1995), Cheung et al. (2013)	Epoxyalkanes	Ethene monooxygenase EtnABCD from *Mycobacterium chubuense* NBB4 Coleman et al. (2006), Coleman et al. (2011a)
	Group 5	Contains 3 protein subunits^b^ (αβ) -C-R encoded by 4 genes Kotani et al. (2003)	Propane, tetrahydrofuran, dioxane, dioxolane, chloroethylether, methyl *tert*-butyl ether, N-nitrosodimethylamine Vainberg et al. (2006), Sharp et al. (2007)	C2-C6 alkanes, tetrahydrofuran, β-hydroxyethoxyacetic acid Kotani et al. (2006), Sales et al. (2013)	Propane monooxygenase PrmABCD from *Gordonia* TY-5 Kotani et al. (2003)
	Group 6	Contains 3 protein subunits^b^ (αβ)-C-R encoded by 4 genes Kotani et al. (2007)	Propane, tetrahydrofuran, dioxane Deng et al. (2018)	propane, butane, tetrahydrofuran, dioxane Kotani et al. (2006)	Propane monooxygenase PrmABCD from *Mycobacterium* TY-6 Kotani et al. (2006)
**CuMMO**: Copper membrane monooxygenases	includes **pMMOs**: Particulate methane monooxygenases	Contains 3 protein subunits (α_3_β_3_γ_3_) encoded by 3 genes Lieberman and Rosenzweig (2004)	C1-C5 alkanes and haloalkanes, C2-C4 alkenes and haloalkenes, C2-C4 ethers Stirling et al. (1979), Burrows et al. (1984), Johnson et al. (2004)	High copper/biomass ratio (pMMO only) Hanson and Hanson (1996), propane, butane, tert-butyl alcohol, C4-C6 ethers Johnson et al. (2004)	Particulate methane monooxygenase PmoCAB from *Methylococcus capsulatus* Bath Stolyar et al. (2001)
**alkB:** integral-membrane non-heme di-iron monooxygenase	alkB, alkM	Contains 3 protein subunits (α_3_)-F-R encoded by 3 genes Smits et al. (2002), Alonso and Roujeinikova (2012)	C_3_-C_13_ alkanes, C_10_–C_20_ alkanes, propylene, 1-butene van Beilen et al. (1994), Johnson and Hyman (2006)	C_5_-C_22_ alkanes Cappelletti et al. (2011), dicyclopropylketone Moreno and Rojo. (2019)	Alkane hydroxylase AlkBFGHJKL from *Pseudomonas putida Gpo1* van Beilen et al. (1994). AlkMa from *Acinetobacter* sp. *M1* Tani et al. (2000), Throne-Holst et al. (2007)
**CYP**: soluble heme-dependent cytochrome P450s	CYP153	Diverse in structure, requires 3 components CYP-F-R Fiorentini et al. (2018)	C_5_-C_16_ alkanes, C_10_-C_30_ alkanes, limonene, cyclohexene, styrene, medium- and long-chain fatty acids van Beilen and Funhoff. (2007)	C_8_-C_16_ alkanes, phytane Liu et al. (2011), Wang and Shao. (2012), Liang et al. (2016a)	Cytochrome P450 alkane hydroxylase from *Alcanivorax dieselolei* Liu et al. (2011)
	Class VII CYP P450	Contains 1 gene consisting with 2 domains; a heme domain and a reductase domain Minerdi et al. (2015)	C_14_-C_16_, C_24_, C_26_ Minerdi et al. (2015)	Medium- and long- chain alkanes Minerdi et al. (2015)	CYP116B5 from *Acinetobacter radioresistens* Minerdi et al. (2015)
**FMO:** Soluble flavin-binding monooxygenase	AlmA	Contains 1 gene Throne-Holst et al. (2007)	C_14_-C_36_ alkanes Throne-Holst et al. (2007), pristane Throne-Holst et al. (2007), Wang and Shao. (2014)	C_18_-C_36_ alkanes, pristane, phytane Wang and Shao. (2012)	AlmA from *Acinetobacter sp.* DSM 17874 Throne-Holst et al. (2007)
	Dioxygenase	Contains 1 gene, requires Cu^2+^ but not NAD(P)H Maeng et al. (1996)	C_10_-C_30_ alkanes, C_12_-C_20_ alkenes, amylbenzene and tridecylbenzene Maeng et al. (1996)	C_10_-C_30_ alkanes Maeng et al. (1996), Sakai et al. (1996)	*Acinetobacter sp.* M1 Sakai et al. (1996)
	LadA	Contains 1 gene consisting of 3 domains; a monooxygenase domain, and 2 NAD(P)H oxidation domains Feng et al. (2007), Tourova et al. (2016)	C_15_-C_36_ Feng et al. (2007), Wang and Shao. (2013), Wang and Shao. (2014), Tourova et al. (2016)	C_22_-C_36_ alkanes Li et al. (2008), Liu et al. (2011)	LadA from *Geobacillus thermodenitrificans* NG80-2 Feng et al. (2007)

a α,β,γ, oxygenase subunits; R, reductase subunit; C, coupling protein; F, ferredoxin; X, protein of unknown function.

b This is tentatively inferred by the homology of the group 5 and group 6 SDIMOs to the group 4 alkene MOs.

There is great interest in detecting and recovering new monooxygenases, due to their interesting catalytic properties and also due to their linkage to useful regulators for biosensor construction. Nested PCR with degenerate primers enabled recovery of novel soluble di-iron monooxygenases (SDIMO) from soils, sediments, and enrichment cultures, and was also useful for identifying interesting isolates which contained multiple SDIMO genes (Coleman et al., 2006). Other PCR approaches have been invaluable for screening isolate collections for AlkB type monooxygenases (Smits et al., 1999; Van Beilen et al., 2003). Metaproteomics approaches have been used to identify novel alkene monooxygenases in enrichments from vinyl-chloride contaminated groundwater (Chuang et al., 2010), and to identify archaeal ammonia monooxygenases (a copper-containing membrane monooxygenase, CuMMO) in marine samples (Morris et al., 2010). Novel CuMMOs have also been isolated from an oilsands tailing pond using stable isotope probing and qPCR methods (Rochman et al., 2020).

### 3.2 Functional Characterisation of Monooxygenases

Once a novel target gene has been identified, it is most important for biosensor development to confirm that the monooxygenase genes are indeed inducible by hydrocarbons (or metabolites thereof) (Vogne et al., 2010). Determining other parameters such as substrate range and kinetics are also very important for related applications like bioremediation. Techniques used to confirm the function of novel monooxygenases may include biochemical assays (resting cells, cell extracts etc.), omics approaches (e.g., transcriptomics, proteomics), or genetic methods (knockouts, knockdowns, heterologous expression).

**FIGURE 2:**
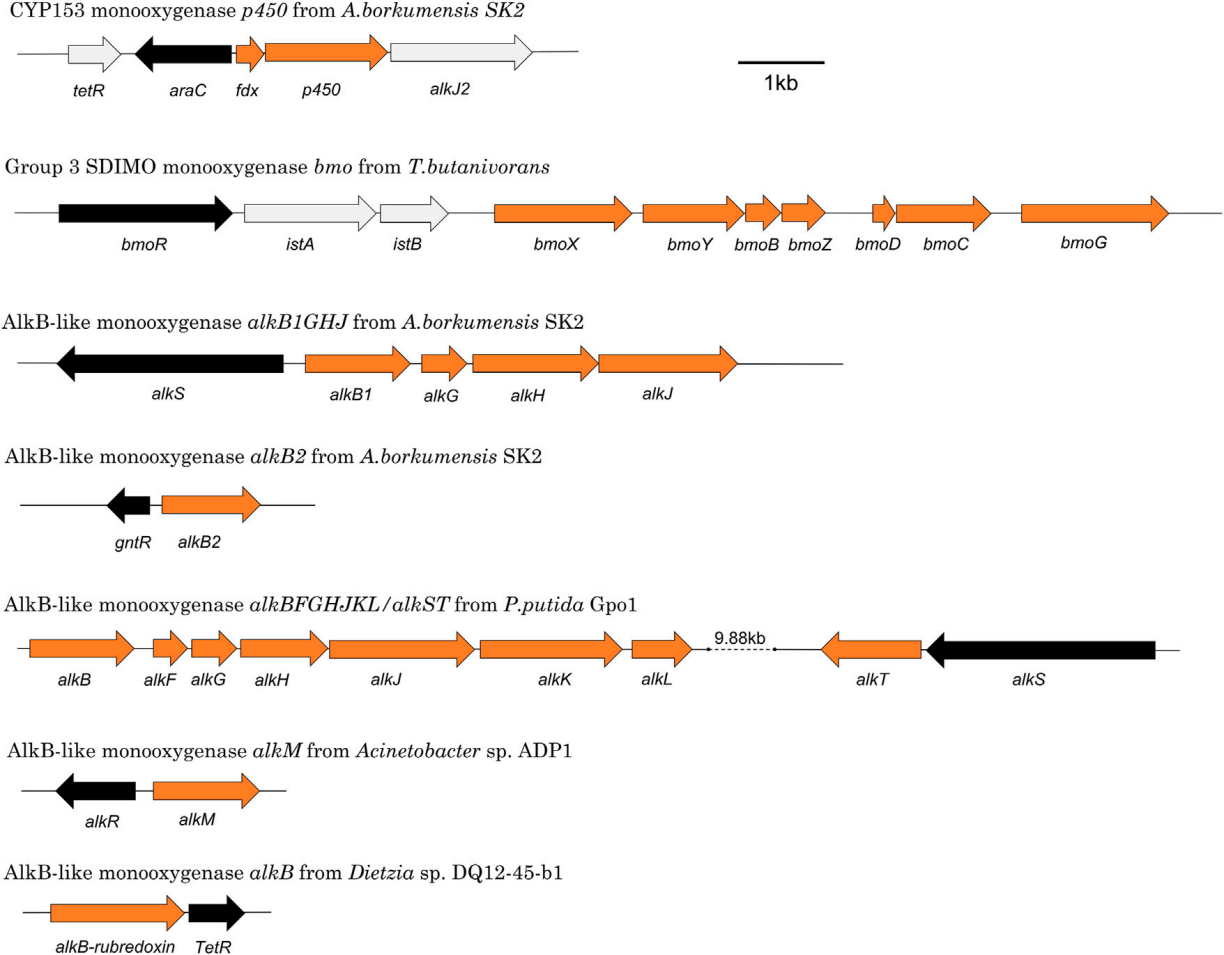
Representative monooxygenase gene clusters showing relative arrangements of metabolic genes (orange) and regulatory genes (black).

Alkane-degrading bacterial species are diverse and some are well-characterised, with Actinobacteria such as *Corynebacterium*, *Mycobacterium, Nocardia* and *Rhodococcus* dominating when gaseous substrates are used (Shennan, 2006) and Proteobacteria such as *Pseudomonas*, *Acinetobacter*, and *Alcanivorax* more typical when liquid substrates are used (Nie et al., 2014a). The best-characterised alkane monooxygenase is AlkB from *P. putida* GPo1, isolated on hexane, which can oxidise C_5_-C_9_ n-alkanes (Baptist et al., 1963; van Beilen et al., 1994). AlkB requires accessory proteins (AlkG, AlkT) to deliver electrons from NADH to enable the activation of molecular oxygen. Many other classes of monooxygenases can also attack alkanes, including iron, flavin, and copper-requiring enzymes (Table 1, also see reference Moreno and Rojo, 2017). The gene arrangements of a selection of characterised alkane monooxygenases can be seen in Figure 2.

There are fewer examples of alkene-oxidising enzymes (Table 1), with the two best-studied systems being the propene monooxygenases of *Rhodococcus rhodochrous* (Gallagher et al., 1997; Gallagher et al., 1998; Smith et al., 1999) and *Xanthobacter* Py2 (Ginkel, 1987; Ginkel et al., 1987; Small and Ensign, 1997; Champreda et al., 2004). Despite having the same primary substrate, these two enzymes are diverse in both sequence and structure; the former is encoded by four genes encoding three enzyme subunits (Smith et al., 1999) while the latter is a six gene, four component system (Small and Ensign, 1997; McCarl et al., 2018). Major advances since the alkene monooxygenases were last reviewed (Ensign, 2001; Shennan, 2006) include the identification, characterisation, and heterologous expression of the genes encoding the ethene monooxygenases (EtnABCD) found in *Nocardioides* and *Mycobacterium* spp. (Coleman and Spain, 2003b; Mattes et al., 2005; Coleman et al., 2011a; McCarl et al., 2018) and the investigation of these enzymes as biocatalysts for epoxide synthesis (Owens et al., 2009; Cheung et al., 2013).

The number of homologs in databases and the number of publications relevant to each representative monooxygenase are shown in Table 2. These numbers reflect the sheer volume of candidate enzymes that have been identified to date; the research at this early stage of the biosensor development framework is abundant. The relationship between monooxygenase genes and hosts is complex and it is likely that the corresponding genes have been subject to extensive lateral gene transfer (Coleman and Spain, 2003a; Das et al., 2015; Minerdi et al., 2015; Liang et al., 2016a; Khadka et al., 2018). The SDIMO and CuMMO type monooxygenases have quite restricted distribution compared to the AlkB and CYP153 enzymes; the latter are common in the genomes of environmental bacteria (Nie et al., 2014b).

**TABLE 2:** Classification and quantification of monooxygenase homologues and related publications in public databases.

Representative enzyme subunit used for BLAST analysis	Monooxygenase homologues			Related publications	
	Uniprot ID	GenBank ID	No. of homologues^a^	Scopus keyword(s)	No. of publications
XamoA, *Xanthobacter* Py2	O87082	AJ006979.1	951	“Propene monooxygenase”	5
MmoX, *Methylococcus capsulatus* Bath	P22869	M90050.3	810	“Soluble methane monooxygenase”	426
EtnC, *M. chubuense* NBB4	D2K2E0	GU174752.1	37	“Ethene monooxygenase”	6
PrmA, *Gordonia* TY5	Q768T5	AB112920	1,551	“Propane monooxygenase”	38
PrmA, *Mycobacterium* TY-6	Q08KF2	AB250938	959		
PmoC1, *Methylococcus capsulatus* Bath	Q603F1	AE017282	1,103	“Particulate methane monooxygenase”	500
AlkB, *P. putida* Gpo1	P12691	AJ245436	5,010	“AlkB”	1855
CYP153, *Alcanivorax dieselolei*	D0Q1H3	GQ980250	5,046	“CYP153”	67
CYP116B5, *A. radioresistens* S13	G9BWN9	HQ685898	2,787	“CYP116B5”	2
AlmA, *Acinetobacter* sp.	AOA2U9IB23	MH357335	5,060	“AlmA” AND “monooxygenase”	14
LadA, *Burkholderia* sp.	A0A095EJX9	CP007785	4,798	“LadA” AND “monooxygenase”	14
AlkMa, *Acinetobacter* sp. M1	Q9AQK2	AB049410	5,012	“AlkMa”	5

a Homologues were defined here as BLAST matches with >40% amino acid identity.

## 4 Identification and Characterisation of Regulatory Systems

### 4.1 Initial Identification of Regulatory System Components

Important questions to be answered for newly discovered regulatory systems include: What are the inducers? Is it a one-component or two-component system? Are there additional layers of control apart from the hydrocarbon (or hydrocarbon metabolite) inducer? The investigation typically begins with bioinformatics, *via* identification of regulator gene(s), and the cognate promoter and operator sequences. Regulator genes are usually identified based on their proximity to the monooxygenase genes, and *via* sequence similarities to known transcription factors. Promoters can be identified on the basis of consensus sequences (e.g., −35 and −10 motifs) and their location upstream of the monooxygenase genes. Operators typically have an inverted repeat structure and will be located proximal to the promoter, either upstream (activators) or downstream (repressors) (Browning and Busby, 2016).

Pull-down assays offer an alternative method of identifying regulator proteins (Ji et al., 2019), in which a DNA containing the promoter sequence is immobilized on beads, cell extracts are washed over the beads, then proteins bound to the promoter sequence can be identified by mass spectrometry. This method allows regulatory proteins to be identified independent of bioinformatic predictions, but it does require that the promoter sequence is known, and it may be complicated by competitive binding of multiple proteins to the promoter; this may cause regulators that bind with lower affinity to be overlooked. Other protein-DNA binding assays useful for identifying promoter/regulator pairs are electrophoretic mobility shift assays (EMSA) and DNase footprinting; in the former case, regulators are detected by their retardation of the gel mobility of a DNA containing the promoter sequence, while in the latter case, they are found by their ability to protect the promoter sequence from DNase digestion (Read et al., 2009).

Vogne et al. (2010) describe five criteria that should be addressed in the characterisation of catabolic regulatory factors, summarised below. In the case of alkane regulatory systems, these criteria have only been met in a handful of cases.

(1) There must be evidence for involvement of the regulator in gene expression.
(2) The genes being controlled must be identified.
(3) The promoter elements associated with the regulatory protein must be identified.
(4) The expression of the regulator gene itself must be investigated.
(5) The inducer compound and its relationship with the regulator must be understood.

### 4.2 Experimental Methods for Characterisation of Regulatory Systems

Two general approaches can be used to confirm the function of regulatory system elements; either untargeted methods e.g., proteomics and transcriptomics, or targeted methods e.g., heterologous expression, knock-ins, knockouts, knockdowns, pull-downs, gel shift assays, or footprinting. These approaches are described below.

Transcriptomics gives information about the expression patterns of different genes under different conditions. If exposure to a suspected inducer leads to higher expression levels of a particular gene, this provides strong preliminary evidence that the enzyme encoded by that gene is part of a metabolic pathway controlled by that inducer; searching upstream from the induced gene then allows discovery of the likely promoter. Alternatively, promoters can be found *via* transcriptomics via pinpointing intergenic regions which are not themselves transcribed. Finally, transcriptomics data can provide clues about the control of the regulatory genes themselves, such as whether these are constitutively expressed, or part of positive or negative feedback loops.

Heterologous expression can be used to confirm the role of regulatory proteins, e.g., by cloning the regulator gene and its cognate promoter and operator elements into a plasmid and adding a reporter gene downstream of the promoter. Exposure to the correct inducer will result in expression of the reporter gene if the combination of regulatory protein, promoter and operator sequences is correct. While a good starting point, there are limitations to this method, as follows; there may be multiple regulatory proteins required to evoke the desired response, the regulatory proteins might be hard to express in a heterologous host due to codon usage issues or due to strain-specific genes required for regulator protein function e.g. chaperones, the structure and type of plasmid used may unexpectedly impact the outcomes due to effects arising from copy number, gene orientation, or transcription read-through.

Gene knock-in methods are another useful targeted approach to testing hypotheses about regulatory systems. This involves integrating a reporter gene downstream of the promoter in the genome of the native organism, either in front of the metabolic genes, or replacing them. Exposure to the correct inducer should yield expression of the reporter gene. Knock-in methods have several advantages over heterologous expression in plasmids, most notably that they sidestep problems arising from codon usage, plasmid copy number, and altered genomic context. The major disadvantage of gene knock-ins is that they may be technically more difficult to generate, depending on the host organism.

Gene knockouts can also be used to interrogate the components of hydrocarbon regulatory systems. Knocking out the putative regulatory gene can be done via homologous recombination (usually replacing the regulator with a resistance gene) or *via* CRISPR-Cas-based methods; the latter are preferable due to the increased specificity and higher frequency of deletion mutants obtainable, but the choice here may be limited by the genetic tools available in the host species of interest. The impact of the knockout on the host organism’s phenotype can be readily tested, e.g., its ability to oxidise hydrocarbons. For repressors, a knockout should yield a constitutive hydrocarbon-oxidising phenotype, while for activators, a decreased or abolished ability to oxidize hydrocarbons would be expected.

Gene knockdowns enable the regulatory gene to be turned down or off temporarily *via* technologies such as interfering RNA (RNAi) (Hannon, 2002; Kim and Rossi, 2018) or dead Cas9 (dCas9) proteins (Dong et al., 2018). The overall strategy here is similar to the knock-outs described above, with the impact tested either via measuring hydrocarbon oxidation in the resultant recombinants or *via* a reporter gene if this has been integrated in place of the metabolic genes. An advantage of the knock-down approach is that it can be used to test inactivation of regulators of essential genes, since the bacteria can be grown first, then the knock-down activated, e.g., testing methane monooxygenase regulators in obligate methanotrophs.

## 5 Regulatory Systems for Alkane Monooxygenases

The most well-studied alkane monooxygenase regulatory systems are those associated with the AlkB and CYP153 monooxygenases, but there is also information available on the regulators of AlkB2, AlkM, AlmA, AlkW, BmoXYBZDC, PrmABCD and SmoABCD. Taken together, these represent nine regulatory systems across seven species that have been experimentally characterised. If putative regulatory proteins identified by sequence analysis only are included, this count increases to 19 systems (Tables 3, 4). Details of the elements of these systems and their functions are described in the sections below. The positioning of a selection of regulatory genes relative to the relevant monooxygenase gene cluster can be seen in Figure 2.

**TABLE 3:** Sequences of characterised alkane monooxygenase promoters.

Promoter name	Species	MO gene	Promoter sequence^a^	References
P_alkB_	*P. putida* Gpo1	*alkBFGHJKL*	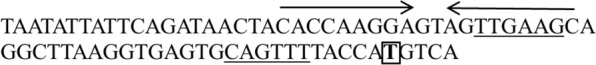	Yuste et al. (1998), Canosa et al. (2000)
P_alkB_	*P. putida* P1	*alkBFGHJKL*	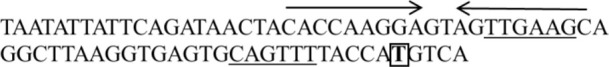	van Beilen et al. (2001)
P_alkB1_	*A. borkumensis* AP1	*alkSB1GHJ*	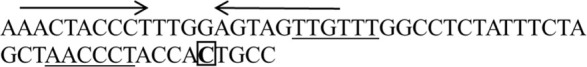	van Beilen et al. (2004)
P_alkB2_	*A. borkumensis* AP1	*alkB2*	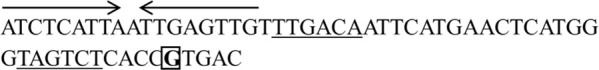	van Beilen et al. (2004)
P_fdx_	*A. borkumensis* SK2	*CYP153 P450-1*	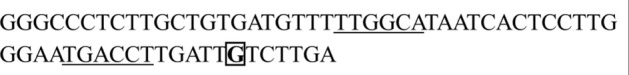	Sevilla et al. (2017)
P_alkB_	*B. cepacia* RR10	*alkB*	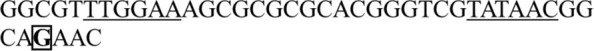	Marín et al. (2001)
P_alkW1_	*Dietzia* sp. *DQ12-45-1b*	*alkW1*	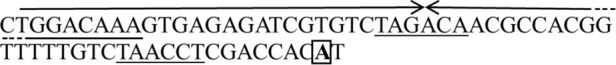	Liang et al. (2016b)
P_alkB1_	*P. aeruginosa* RR1	*alkB1*	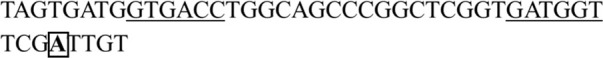	Marín et al. (2003)
P_alkB2_	*P. aeruginosa* RR1	*alkB2*	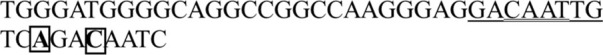	Marín et al. (2003)
P_fdx_	*Dietzia* sp. *DQ12-45-1b*	*CYP153*	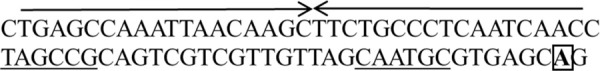	Liang et al. (2016a)
P_alkM_	*Acinetobacter* ADP1	*alkM*	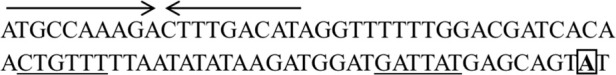	Ratajczak et al. (1998a)
P_*alkB2*_	*P. aeruginosa* SJTD-1	*alkB2*	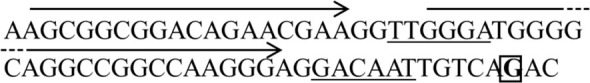	Ji et al. (2019)
P_prm_	*Rhodococcus* sp. BCP1	*prmABCD*	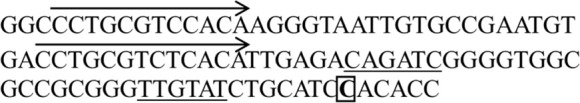	Cappelletti et al. (2015)
P_smo_	*Rhodococcus* sp. BCP1	*smoABCD*	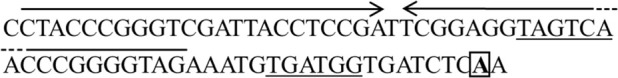	Cappelletti et al. (2015)
P_alkB_	*Rhodococcus* sp. BCP1	*alkB*	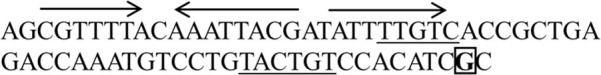	Cappelletti et al. (2011)

a The −35 and −10 motifs are underlined, transcription start points are in bold, and inverted repeats are shown with arrows. All of these elements were identified in prior studies except in the case of P_alkB1_ of *A. borkumensis* AP1, where we have tentatively identified the −35, −10, and start point as part of this study.

**TABLE 4:** Summary of identified and/or characterised monooxygenase regulatory systems from literature.

Regulator family	Nature of regulator	Regulator gene	Cognate promoter^a^	Bacterial strain	Monooxygenase	Inducers	Evidence for regulator function	References
LuxR/MalT	Activator	*AlkS*	P_alkB_	*P. putida* GPo1	*alkBFGHJKL/alkST*	C_6_-C_10_ n-alkanes	Heterologous expression in *E.coli*	Sticher et al. (1997), Panke et al. (1999), Canosa et al. (2000), van Beilen et al. (2001)
			P_alkB_	*P. putida* P1	*alkBFGHJKL/alkST*		Inferred from results with GPo1	van Beilen et al. (2001)
	Activator		P_alkB1_	*A. borkumensis* SK2	*alkSB1GHJ*	C_5_-C_12_ n-alkanes	Sequence analysis only	Schneiker et al. (2006)
			P_alkB1_	*A. borkumensis* AP1	*alkSB1GHJ*	C_5_-C_12_ n-alkanes	S1 nuclease protection assay; *lacZ* transcriptional fusion	van Beilen et al. (2004)
AraC/XylS	Activator	*cypR*	*P_fdx_	*A. borkumensis* SK2	*CYP153*	C_8_-C_18_ n-alkanes	Promoter-GFP transcriptional fusions; gene-inactivation	Schneiker et al. (2006), Sevilla et al. (2017)
	Activator		*P_fdx_	*Dietzia* sp. *DQ12-45-b1*	*CYP153*	C_8_-C_14_ n-alkanes	Promoter-*lacZ* fusion assays; gene-inactivation; RACE analysis	Liang et al. (2016b)
	Activator	*alkR*	*P_alkM_	*Acinetobacter* sp. ADP1	*alkM*	C_7_-C_18_ n-alkanes	Gene-inactivation; *lacZ* chromosomal fusions	Ratajczak et al. (1998a), Ratajczak et al. (1998b)
	Activator	*alkRa*	*P_alkMa_	*Acinetobacter* sp. M1	*alkMa*	>C_22_ n-alkanes	Sequence analysis only	Tani et al. (2000)
	Activator	*alkRb*	*P_alkMb_	*Acinetobacter* sp. M1	*alkMb*	C_16_-C_22_ n-alkanes	Sequence analysis only	Tani et al. (2000)
	—	*Orf1*	*—*	*A. dieselolei* B5	*alkB2*	C_12_-C_26_ n-alkanes	Sequence analysis only	Liu et al. (2011)
	*—*	*Orf3*	*—*	*A. dieselolei* B5	*CYP153*	C_8_-C_16_ n-alkanes	Sequence analysis only	Liu et al. (2011)
None	Repressor	*almR*	*—*	*A. dieselolei* B5	*almA*	C_22_-C_30+_ n-alkanes	Gene-inactivation experiments	Liu et al. (2011), Wang and Shao (2014)
TetR	Repressor	*alkX*	P_alkW1_	*Dietzia* sp. *DQ12-45-b1*	*alkW1X*	C_10_-C_24_ fatty acids	Dnase I footprinting assay, EMSA	Liang et al. (2016a)
	*—*	*Orf10*	*—*	*A. hongdengensis A-11-3*	*alkB1*	C_12_-C_24_ n-alkanes	Sequence analysis only	Wang and Shao (2012)
GntR	Repressor	*gntR*	P_alkB2_	*A. borkumensis* SK2	*alkB2*	C_8_-C_16_ n-alkanes	Sequence analysis only	van Beilen et al. (2004), Schneiker et al. (2006)
	*—*	*Orf20*	*—*	*A. hongdengensis A-11-3*	*alkB2*	C_12_-C_24_ n-alkanes	Sequence analysis only	Wang and Shao (2012)
	*—*	PA1526	P_alkB2_	*P. aeruginosa RR1/PA O 1*	*alkB2*	C_12_-C_20_ n-alkanes	Sequence analysis only	Marín et al. (2003), Smits et al. (2003)
LysR	Repressor	CrgA	P_alkB2_	*P. aeruginosa* SJTD-1	*alkB2*	C_14_-C_20_ n-alkanes	Gene inactivation; EMSA; DNase I footprinting; promoter-GFP plasmid assays	Ji et al. (2019)
σ^54^—dependent	Activator	*bmoR*	P_bmo_	*T. butanivorans*	*bmoXYBZDC*	C_2_-C_8_ n-alkanols	Gene-inactivation; promoter-lacZ fusion assays	Kurth et al. (2008)
Fis	*—*	*—*	*P_prm_	*Rhodococcus* sp. BCP1	*prmABCD*	C_3_-C_4_ n-alkanes	Sequence analysis only	Cappelletti et al. (2015)
	*—*	*—*	*P_prm_	*Rhodococcus* sp. RHA1	*prmABCD*	*—*	Sequence analysis only	Cappelletti et al. (2015)
	*—*	*—*	*P_prm_	*Rhodococcus opacus* PD630	*prmABCD*	*—*	Sequence analysis only	Cappelletti et al. (2015)
	*—*	*—*	*P_prm_	*M. smegmatis* MC2 155	*prmABCD*	*—*	Sequence analysis only	Cappelletti et al. (2015)
LuxR + NarQ-like sensor kinase	*—*	*—*	*P_smo_	*Rhodococcus* sp. strain BCP1	*smoABCD*	C_1_-C_7_ n-alkanes	Sequence analysis only	Cappelletti et al. (2015)
	*—*	*—*	*P_smo_	*M. chubuense* NBB4	*smoABCD*	*—*	Sequence analysis only	Coleman et al. (2011a), Cappelletti et al. (2015)

a Asterisks here indicate that the promoter has not been named in previous reports. Promoter names assigned here are based on previous naming conventions.

It is important to note that the well-characterised alkane regulatory systems represent only a small fraction of the total diversity, and comparisons between these indicate the limitations in extrapolating conclusions from one system to another. The bias in research towards AlkB and CYP153 has left large knowledge gaps for other systems, and a further limitation is that more work has been focused on the monooxygenases rather than their regulatory systems—this is a major bottleneck in the development of alkane biosensors. A better understanding of the regulation of alkane oxidation systems is needed, *via* following the characterisation criteria (Vogne et al., 2010) outlined in the previous section.

### 5.1 Promoters and Operator Sequences

Identifying promoter and operator elements is an essential part of understanding the mechanism of action of transcription factors. Sigma factors are key players in transcriptional initiation and in bacteria σ^70^ and σ^54^ are the two dominant subclasses. Each recognizes and binds to specific promoter elements that allows for the recruitment and correct coordination of RNA polymerase. Promoters can be identified by locating either the σ^70^ promoter elements at the −10 and −35 positions relative to the transcriptional start site (Paget and Helmann, 2003), or the σ^54^ promoter elements at the −12 and −24 positions (Buck and Cannon, 1992; Francke et al., 2011). The known promoters and operator sequences associated with alkane monooxygenases are summarised in Table 3. The degree of characterisation of these elements varies across species and there is much work still to be done to understand the details of promoter-protein relationships in the hydrocarbon metabolism regulators.

The operator elements associated with monooxygenase promoters consist of repeat sequences adjacent to the −35 site. Nearly all the monooxygenase promoter sequences identified in literature show such repeats (Table 3). Sometimes these sequences are imperfect direct repeats, like the sequence enabling CrgA binding in the *alkB2* promoter of *P. aeruginosa* SJTD-1 (Jiménez et al., 2019). In other cases, these are inverted repeats, such as that found between *alkR* and *alkM* in *Acinetobacter* sp. ADP1; interestingly in this case, the regulator protein is in the AraC/XylS family, which are normally associated with direct repeats (Ratajczak et al., 1998a).

The identification of operator sequences provides crucial insights into the function of regulatory systems. For example, the operators recognised by the AlkS regulator in *P.putida* Gpo1 are found upstream of both the monooxygenase promoter P_alkB_ and also the AlkS promoter P_alkS2_ (Canosa et al., 2000), indicating a positive feedback loop in this system. The inverted repeat sequence recognised by AlkS is highly homologous to other operators controlled by LuxR-family proteins (Fuqua et al., 1996). Gene expression from the P_alkB_ promoter drops to negligible levels when the operator is removed (Canosa et al., 2000), confirming that AlkS is an activator protein rather than a repressor. The function of the AlkS operator was confirmed in an assay using a recombinant *E. coli* containing a chromosomal *xylE* reporter under the control of *alkS* and *P*_*alkB*_ (van Beilen et al., 2001). A reduction in expression of *xylE* was seen when the operator sequences were supplemented on a plasmid, consistent with competition for AlkS between the plasmid and chromosomal sequences (van Beilen et al., 2001).

Although the presence of repeat sequences near a promoter is indicative of an operator, this needs to be experimentally validated, even in cases where homology to characterised operators is high. The promoters of both *alkB1* and *alkB2* in *A.borkumensis* contain homologs of the AlkS operator from *P.putida* Gpo1. While a transcriptional fusion of *P*_*alkB1*_ to lacZ was responsive to alkanes, a similar fusion to *P*_*alkB2*_ while not (van Beilen et al., 2004), implying that the putative AlkS binding site upstream of *alkB2* is not functional, despite its strong homology to functional operator sequences.

### 5.2 Understanding Expression of Regulatory Genes, and the Inducer-Protein Relationship

Most alkane monooxygenase regulators are activator proteins (Moreno and Rojo, 2019), induced by the alkane directly, e.g. AlkS in *P. putida* Gpo1 (Kok et al., 1989), or induced by a downstream metabolite, e.g. BmoR in *T. butanivorans* (Kurth et al., 2008)*.* The latter systems depend on the monooxygenase having a non-zero level of expression in the “switched off” state, so the inducer can be made from the alkane. Most alkane regulatory systems display self-regulation, such as AlkS in *P. putida* Gpo1. In the absence of alkanes, *alkS* is expressed from P_alkS1_, and expression levels are kept low by a self-repressive effect of AlkS on P_alkS1_. Upon the addition of alkanes, AlkS activates the adjacent P_alkS2_ promoter, driving high levels of AlkS expression in a positive feedback loop, and also repressing expression from P_alkS1_ (Canosa et al., 2000). In *Dietzia* sp. *DQ12-45-b1*, fatty acids reduce AlkX repression on the *alkW1* promoter in a positive feedback loop (Liang et al., 2016b). Meanwhile, in *A. borkumensis* AP1, the regulatory gene a*lkS* is expressed constitutively, independent of the presence of alkanes (van Beilen et al., 2004).

Species that contain multiple monooxygenases complicate the understanding of induction processes due to possible overlaps between the inducer range and substrate range of the monooxygenases. For example, in *A. dieselolei* B5, the CYP153 is expressed in the presence of C_8_-C_16_ n-alkanes, the *almA* monooxygenase is induced by C_22_-C_36_ n-alkanes, both *alkB1* and *alkB2* monooxygenases are induced by C_12_-C_26_ n-alkanes, and *alkB1* and *almA* expression can also be upregulated by the branched alkanes pristane and phytane (Liu et al., 2011). An overlapping substrate range for two AlkB-type monooxygenases is also seen in *A. borkumensis* AP1, where C_10_, C_12_, C_14_ and C_16_ *n*-alkanes induce both *alkB1* and *alkB2* (van Beilen et al., 2004). The correlations of inducers to regulators can be teased apart *via* the generation of deletion mutants, but this is not always straightforward, e.g., in situations where one monooxygenase can generate the metabolite inducer for another.

### 5.3 Alkene/Alkane Specificity

The AlkM monooxygenase of *A. baylyi* ADP1 is an alkane-induced system activated by the AlkR regulator (Ratajczak et al., 1998b). However, AlkR also responds strongly to the alkene octadecene, which is second only to octadecane in its strength as an inducer (Zhang et al., 2012b). The shorter alkene dodecene is also a very effective inducer for the ADP1 AlkR regulator. These findings emphasise the fact that the size of the inducer molecule is more important than the presence of a double bond in determining whether it will act as an effective inducer. It is likely that other “alkane-inducible” regulators also respond to similarly-sized alkenes, regardless of the substrate range of the cognate monooxygenase. This is part of the relationship between inducer compounds and regulatory proteins that should be explored further.

### 5.4 Structures of Alkane Regulators

Transcriptional regulators have two protein domains—a DNA-binding domain, and a sensing domain. DNA-binding domains are readily identifiable because they contain conserved motifs, such as helix-turn-helix and zinc finger domains (Harrison, 1991), while sensing domains are more diverse and hard to identify based solely on bioinformatics. Diverse families of transcription factors have been recruited as alkane sensors, including proteins from the LuxR, AraC, TetR, GntR, LysR and Fis families (Table 4). Each of these families have unique organisation and features, e.g., LuxR and AraC family proteins have the DNA-binding motif at the C-terminus, while GntR and TetR family proteins have the DNA-binding domain at the N-terminus (Gallegos et al., 1997; Schrijver et al., 1999; Santos et al., 2012; Cuthbertson and Nodwell, 2013). There is also great diversity within families, e.g., the AlkR and CypR regulators of *Acinetobacter* sp. ADP1 and *Dietzia* sp. DQ12-45-b1 have sequence motifs common to AraC/XylS family proteins, but the overall sequence identity between these two proteins is only 26% (Liang et al., 2016a).

The AraC/XylS regulators also have different inducer ranges even within the same species. This is particularly noticeable when comparing the *alkR*, *alkRa* and *alkRb* candidates from *Acinetobacter* sp. APD1 and M1. There are three distinct inducer ranges for these monooxygenases, suggesting no such pattern exists at the regulatory family level. In some cases, however, there are similarities in the inducer range of each family of regulators. The inducers for the characterised LuxR-MalT family regulators are highly consistent even across bacterial species. This could be another way to infer characteristics of uncharacterised regulatory systems. For example, the regulator O*rf3* linked to the *CYP153* cluster from *A. dieselolei* B5 has an overlapping inducer range to *cypR* from both *A. borkumensis* SK2 and *Dietzia* sp. *DQ12-45-b1* suggesting it might have a similar mechanism of action.

There is very little research on the protein structure of alkane regulators, and how this determines the relationships with inducers. Only one crystal structure is available, for AlkX from *Dietzia* sp. DQ12-45-1b (Liang, 2017). This protein appears to represent a new sub-family of TetR regulators (Liang, 2017). The AlkX binding pocket can accommodate fatty acids, which supports previous data showing that C_10_-C_24_ fatty acids interfere with DNA binding of this regulator (Liang et al., 2016b); this feature was confirmed by the finding that AlkX crystals made via heterologous expression in *E. coli* contained host-derived palmitic acid in the substrate-binding pocket. The operator associated with AlkX is longer than typical sequences, consistent with AlkX binding as a dimer or pair of dimers. The crystal structure of AlkX gives insight into the inducer range, with the arrangement of hydrophobic and hydrophilic residues in the binding pocket suggesting a minimum chain length of fatty acid required to remain in the pocket.

### 5.5 Differences in Regulation of AlkB-Like Monooxygenases

Given the diversity in sequences and bacterial hosts of AlkB monooxygenases (Nie et al., 2014a), it is not surprising that the regulation of these systems is also diverse. In most cases, the *n*-alkane is the inducer, however in at least one case (*Rhodoccocus* sp. BCP1), an alcohol can also act as inducer (Cappelletti et al., 2015). Comparison of the regulation of the ADP1 and GPo1 AlkM and AlkB enzymes is informative (note that the ADP1 AlkM monooxygenase is a homolog of AlkB, at 41% amino acid identity). Regulation of AlkM in ADP1 is simple, and occurs solely *via* the AlkR protein, which is constitutively expressed at low levels in the cell, and activates *alkM* expression in the presence of long-chain alkanes (Ratajczak et al., 1998a). In contrast, the situation in *P. putida* GPo1 is more complex (Yuste et al., 1998), with the *alkBFGHJKL* cluster controlled by the AlkS regulator *via* a positive feedback loop, as described in a previous section. The regulator AlkS is partly responsible for limitations on the hydrocarbon substrate range of *P. putida* GPo1, since the range of inducers that it recognises is narrower than the oxidation range of AlkB.

Differences in the regulation of AlkB-type monooxygenases have implications for future biosensor development. Ratajczak et al. (1998b) observed that while both medium- and long-chain n-alkanes (C_7_-C_18_) induce *alkM* transcription in *Acinetobacter* sp. ADP1, the organism can only grow on larger alkanes (>C_12_), and this induction pattern was confirmed in an ADP1 AlkR-based biosensor (Zhang et al., 2012a). Interestingly, the opposite pattern of inducers vs. growth substrates is seen in GPo1. Shingler (2010) refers to these situations as “regulatory bottlenecks” for catabolic performance, and this should be kept in mind when investigating new bacterial isolates for development of hydrocarbon biosensors, i.e., the range of compounds a strain can utilise may not match the inducer range of its regulatory proteins. These phenomena are not unique to AlkB regulation; e.g., in *Rhodococcus* sp. BCP1, transcription of *smoABCD* is induced by methane despite BCP1 being unable to use methane as a carbon source (Cappelletti et al., 2015).

Comparison of the AlkS regulators in *P. putida* GPo1 and *A. borkumensis* AP1 emphasises the diversity in bacterial hydrocarbon-sensing systems. There is 32% sequence identity between these two AlkS proteins, both contain a helix-turn-helix DNA binding domain, and both recognise a binding site upstream of the promoter which contains a 20 bp inverted repeat; this motif is common to LuxR family regulators (Santos et al., 2012). The AlkS protein from *P. putida* GPo1 can cross-activate expression of the *alkB1* gene in *A. borkumensis* (van Beilen et al., 2004), suggesting that the helix-turn-helix site in the proteins and the cognate operator element in both species are functionally comparable. Despite these similarities, there are also many differences in the function of these two AlkS regulators, such as the constitutive vs. positive feedback mode of control of the regulator gene (see above section), and the presence in *P. putida* GPo1 of an additional inhibitory global regulation network *via* Hfq and Crc (Moreno and Rojo, 2017). Understanding the expression patterns of the regulatory proteins themselves is a key part of characterising the regulatory system as a whole.

### 5.6 Limitations of Sequence Analysis

Bioinformatic analysis of open reading frames in proximity to monooxygenase genes can be used to identify putative regulatory genes. For example, a gene upstream of the *alkB2* monooxygenase in *A. borkumensis* SK2 encodes a GntR homolog, so a logical hypothesis might be that this GntR homolog is the regulator of the monooxygenase. More weight is added to this hypothesis when further analysis reveals that *A. hongdengensis* A-11-3, *P. aeruginosa RR1*, and *P. aeruginosa* SJTD-1 also contain GntR-like proteins in the same position relative to their *alkB2* genes (Wang and Shao, 2012; Liu et al., 2014)*.* However, further work in strain SJTD-1 surprisingly revealed that CrgA, a LysR-type regulatory protein, was actually the regulator of the *alkB2* gene, despite the fact that this regulator is >300 kb away from the monooxygenase gene in the genome (Ji et al., 2019). This result was validated using knock-out, EMSA, footprinting, and promoter-probe assays, but it is not involved in the monooxygenase regulation.

### 5.7 Added Complexity: Further Layers of Regulation of Monooxygenase Expression

The transcription factors that respond to alkanes or their metabolites are not the only players in the regulation of alkane oxidation genes. Aside from methanotrophs and a few other obligate hydrocarbonoclastic bacteria, most hydrocarbon-assimilating species isolated to date are heterotrophic generalists, and do not preferentially utilise n-alkanes. Therefore, confirming the absence of other more easily-utilised carbon sources is just as important for these bacteria as sensing the presence of the hydrocarbon. This is managed by catabolite repression (Moreno and Rojo, 2019). There is evidence that chemotaxis towards alkanes and alkane uptake are also strongly linked to the initial sensing and subsequent metabolism of alkanes in *A. dieselolei*, further reflecting that the expression of regulatory proteins for hydrocarbon detection can be influenced by, and linked to, other cellular functions (Moreno and Rojo, 2019).

Catabolite repression has been well-studied in *P. putida* GPo1, where the alkane degradation pathways are repressed by succinate, lactate, pyruvate or rich complex media like LB (Yuste et al., 1998; Dinamarca et al., 2003). Similarly, the *prmA* and *smoA* monooxygenases of *Rhodococcus* sp. BCP1 are repressed by succinate, glucose or LB medium in the presence of alkanes (Cappelletti et al., 2015), although AlkB from the same strain is not affected in the same way (Cappelletti et al., 2011). In *B. cepacia* RR10, glucose, arabinose, lactose and fructose repress *alkB* expression in the presence of the inducer tetradecanol (Marín et al., 2001). Interestingly, catabolite repression is not seen when *alkB* from *P. putida GPo1* is heterologously expressed in *E. coli*, implying differences in these mechanisms between species (Staijen et al., 1999). This aligns with the fact that the preferred carbon sources of the two differ—glucose for *E. coli*, organic acids for *Pseudomonas*. It is likely that similar global catabolite repression systems exist in other facultative hydrocarbon oxidisers that are less well-characterised.

Product repression is another layer of control of expression of monooxygenases, and this can be exerted either directly or indirectly. In the case of the butane monooxygenase of *T. butanivorans*, fatty acids generated by the butane degradation pathway (typically butyrate) directly bind to and repress the monooxygenase (Doughty et al., 2006). Fatty acids also repress CYP153 in *Dietzia* sp. DQ12-45-1b (Liang et al., 2016a) and *alkB* in *B. cepacia* RR10 (Marín et al., 2001). The ability of fatty acids to repress alkane oxidation pathways has most likely evolved to prevent potential toxicity from the accumulation of such products (Moreno and Rojo, 2019).

Growth phase can also impact the regulation of monooxygenase-mediated pathways. Expression of the *alkB* monooxygenase in *P. putida* Gpo1 was significantly decreased when cells entered stationary phase compared to exponential phase (Yuste et al., 1998). This trend is also seen in *A. borkumensis* strains AP1 and SK2, where expression of *alkB1* and *alkB2* decreased in stationary phase (Schneiker et al., 2006). The opposite is seen with *alkB* expression in *B. cepacia* RR10 where detectable transcripts were highest at early stationary phase (Marín et al., 2001). Meanwhile, in *P. aeruginosa* PAO1, *alkB1* is strongly expressed in late exponential phase while *alkB2* is expressed more in early exponential phase (Marín et al., 2003). The molecular mechanisms behind these growth-phase dependent differences in regulation in these different species are unclear.

## 6 Regulatory Systems for Alkene Monooxygenases

The enantioselectivity of epoxidation by alkene monooxygenases makes them valuable in the production of pharmaceutical precursors and other fine chemicals (Owens et al., 2009; Cheung et al., 2013). For this reason, research thus far has focused on the monooxygenase itself, leaving large gaps in our understanding of the regulation of these systems. An important distinction between the alkene and alkane monooxygenases is the fact that while alkane monooxygenases show activity on both alkanes and alkenes, and their regulatory proteins often accept both kinds of substrates if the carbon chain length is in the right range, the reverse is not true, and alkanes are generally not good substrates or inducers for alkene monooxygenases (Zhou et al., 1999). Because far fewer alkene-oxidising systems have been studied, the sections below are organised differently to the corresponding material on alkane regulation above, and we have taken a case-by-case approach, rather than attempting to draw general conclusions across all the systems.

**FIGURE 3:**
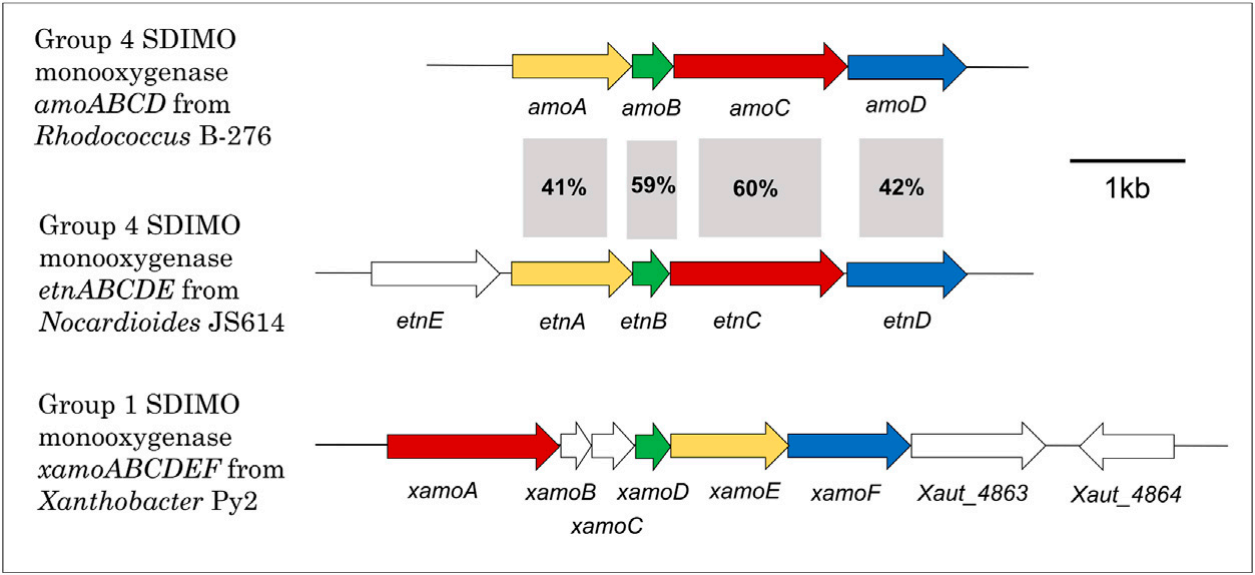
Alkene monooxygenase gene cluster configurations in *Xanthobacter* Py2, *Rhodococcus* B-276 and *Nocardioides* JS614. Colours indicate type of gene in monooxygenase to aid with comparison between clusters. Yellow = beta subunit; green = coupling protein; red = alpha subunit; blue = rubredoxin; white fill = all other genes. % identity also presented.

Figure 3 shows the gene configuration of the two cases discussed below—*Xanthobacter autrophicus* Py2 (XamoABCDEF/Xamo) and *Nocardioides* sp. JS614 (EtnABCD/EtnMO). The shared subunits in these monooxygenases are 23–28% identical to each other, and it is clear the gene arrangement between the two is quite different. The sequence identity of each subunit is also compared to the *amoABCD* cluster from *Rhodococcus rhodochrous* B276, an archetypal alkene monooxygenase, for reference. As shown in the figure, the sequence identities between the B276 and JS614 subunits are much higher, between 41 and 60%. This emphasizes the similarities in both organization and sequence in Actinobacterial clusters. The sequence identities between B276 and Py2 subunits were only 23–32%. The regulation of the *R. rhodochrous* monooxygenase has not been characterized and so won’t be examined in detail in this review.

### 6.1 Regulation of Alkene Monooxygenase in *Xanthobacter autotrophicus* Py2

The alkene monooxygenase of *Xanthobacter* Py2 (XamoABCDEF/Xamo) is a 6-component enzyme that belongs to SDIMO group 1 (Figure 3; Table 1). Strain Py2 was isolated on propene and Xamo was originally identified as a propene-oxidising enzyme (Ensign, 1996), but it also attacks aromatic compounds, and these can support the growth of Py2 (Zhou et al., 1999). One challenge in understanding how Xamo is regulated is uncertainty surrounding the nature of the inducer—it could be the alkene or an epoxide metabolite (Ensign, 1996); resolving this issue is tricky since the wild-type host will rapidly convert alkenes to epoxides, and to date, no effective heterologous expression systems have been reported either for the monooxygenase or the regulators. Early work on this system by Ensign (1996) showed that Xamo induction was possible even in cells grown on glucose, although Small and Ensign later reported that catabolite repression of Xamo occurred during growth on other carbon sources (Small and Ensign, 1997).

A promoter upstream of the XamoA subunit has been identified via sequence analysis, which is 60% identical to the sigma54-dependent promoter consensus sequence (Zhou et al., 1999). This is likely to be the promoter involved in the regulation of the monooxygenase expression.

Analysis of cosmid libraries provided evidence that the control of the Py2 propene monooxygenase might be part of a larger system. Complementation of mutants of Py2 with cosmid clones restored growth on epoxypropane, but interestingly, this activity became constitutive. This indicated that the 22 kb section of DNA in the cosmid contained the metabolic genes but not the necessary regulatory genes (Swaving et al., 1995), and further implied that the system was under at least one layer of negative regulation. The fact that the Py2 Xamo can also oxidise benzene, toluene and phenol suggests that this monooxygenase could be under long range control typical of aromatic hydrocarbon monooxygenases (Zhou et al., 1996).

A later shotgun proteomics study identified a candidate transcriptional regulator for the Py2 monooxygenase (Xaut_4864), that is encoded by a gene located between the Xamo gene cluster and the epoxide carboxylase gene cluster (Broberg and Clark, 2010). Xaut_4864 has a DNA binding domain similar to the MerR family of regulators but is missing 16 of 32 conserved residues typically found in MerR proteins, including the critical Cys82, and thus Xaut_4864 is thought to behave differently to MerR proteins in that it most likely does not need a metal ion to function. Xaut_4864 was identified as a protein expressed only in propylene-grown cells and thus is likely to be an activator of Xamo expression that also induces its own expression.

### 6.2 Regulation of Alkene Monooxygenase in *Nocardioides* sp. JS614

The alkene monooxygenase of *Nocardioides* JS614 (EtnABCD/EtnMO) is quite different from that of Py2; this is a 4-component enzyme, part of SDIMO group 4 (Figure 3; Table 1), and enables growth of the host on ethene and vinyl chloride. Despite being the first ethene-oxidising bacterium to be fully genome-sequenced (Coleman et al., 2011b), the regulatory mechanisms surrounding alkene degradation in *Nocardioides* sp. JS614 are still unclear. Early work on this organism revealed an unusual starvation response (Mattes et al., 2005; Chuang and Mattes, 2007), in which alkene-starved cultures or cultures pre-grown on acetate exhibited long lag periods before growth on alkenes recommenced. A key finding was that addition of ethene oxide (epoxyethane) eliminated the lag periods, suggesting that the epoxide was the inducer, and that the starvation response was due to the bacteria being initially unable to generate this inducer from the alkene substrate.

Peptide mass fingerprinting identified seven proteins in JS614 cells expressed in the presence of vinyl chloride, epoxyethane and ethene (Chuang and Mattes, 2007); these were all identified as components of the monooxygenase or putative downstream metabolic enzymes (e.g., dehydrogenases and transferases) but no regulatory proteins were detected. Inspection of the genome sequence of JS614 reveals that a two-component sensing system is encoded immediately adjacent to the alkene catabolic genes (see Figure 3 in Mattes et al., 2010); this consists of a CdaR family transcription activator and a DmcR family sensor kinase. Homologs of these regulators are also found adjacent to the alkene metabolic genes in other ethene-oxidising bacteria, but there is only preliminary experimental evidence to date to confirm their functions (see below Section 7).

Another clue about regulation of alkene oxidation in JS614 comes from Taylor et al. (2010), who found that the addition of ethene oxide to cultures allowed expansion of the growth substrate range to include propene and butene, which are normally cometabolised, but cannot support growth. These findings reinforce the hypothesis developed from earlier work that the epoxide is the inducer of the monooxygenase in JS614, and also highlight again the important distinction between inducers of regulatory proteins and substrates of catabolic enzymes. Understanding this distinction is critical to the successful development of biosensors and emphasises the importance of characterisation of regulatory proteins themselves, rather than trying to ascertain inducers based solely on the substrates of the monooxygenase.

## 7 *Mycolicibacterium chubuense* NBB4: A Case Study of Diverse Alkane and Alkene Monooxygenases and Regulators

**FIGURE 4:**
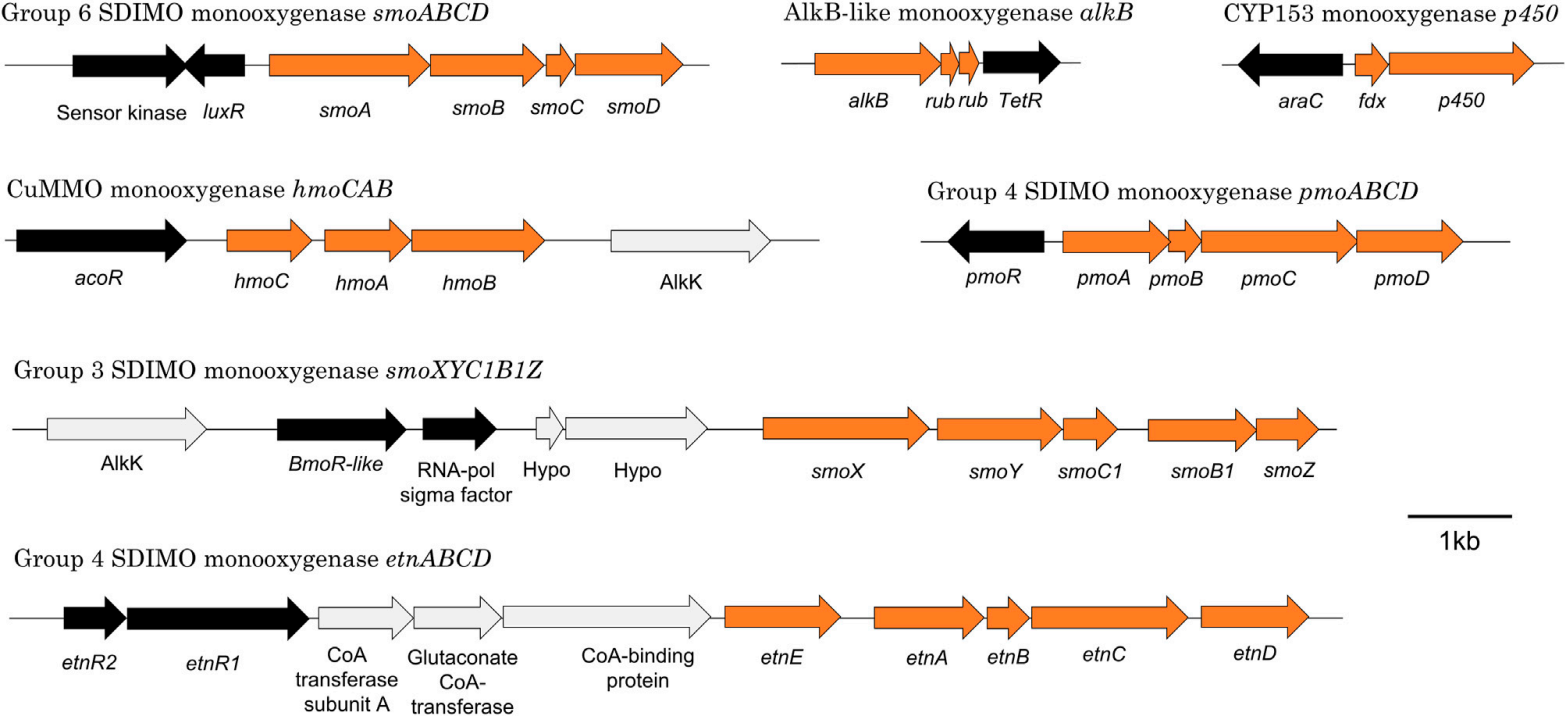
Organisation of seven representative monooxygenase gene clusters from *M. chubuense* NBB4. Orange arrows represent monooxygenase subunits, black arrows represent putative regulator genes, and light grey arrows show other genes in the region.

*Mycolicibacterium chubuense* NBB4 (Coleman et al., 2006) is a hydrocarbon degrader originally isolated on ethene that can grow on many alkanes and alkenes, and is capable of co-metabolism of vinyl chloride and 1,2-dichloroethane (Coleman et al., 2011a). These activities are attributable to the diverse monooxygenases in NBB4 cells, including SDIMOs, AlkB, p450, and CuMMO (Table 5). Coordination of the activities of all these monooxygenases must involve complex regulation, which is thus far not understood. However, the availability of a genome sequence has allowed identification of the likely regulatory genes associated with each monooxygenase gene cluster (Figure 4; Table 5), and generation of hypotheses about their functions, as described below.

**TABLE 5:** Putative regulatory genes associated with monooxygenase clusters in *Mycobacterium chubuense* NBB4.

Gene cluster	MO class	Substrates	Putative regulatory gene(s)	Regulatory protein family	References
*smoXYB1C1Z*	SDIMO Group 3	C_2_-C_4_ alkanes and alkenes	MYCCH_RS28740	AcoR; CadC1 HTH domain	Martin et al. (2014)
			MYCCH_RS28735	SigC-type transcription factor	
*pmoABCD*	SDIMO Group 4	Propene^a^	MYCCH_RS26755	CdaR; GAF domain	Coleman et al. (2011a)
*hmoCAB*	CuMMO	C_2_-C_4_ alkanes and alkenes	MYCCH_RS28775	AcoR; Fis HTH domain and PEP-CTERM-box	Coleman et al. (2012)
*smoABCD*	SDIMO Group 6	Propane^a^	MYCCH_RS26425	LuxR; REC and HTH domains	Coleman et al. (2011a)
			MYCCH_RS26430	Histidine kinase; GAF domain	
*etnABCD*	SDIMO Group 4	C_2_-C_8_ alkenes and chlorinated alkenes	EtnR1; MYCCH_RS29055	CdaR; PucR HTH domain	Coleman et al. (2011a), Moratti et al. (2016)
			EtnR2; MYCCH_RS29050	DmcR; MEDS domain	
*CYP153*; *fdx-cyp-fdr*	Cytochrome P450	C_5_-C_14_ alkanes^a^	MYCCH_RS28400	AraC	Coleman et al. (2011a)
			MYCCH_RS28420	TetR	Coleman et al. (2011a)
*alkB-rubA1-rubA2*	alkB	C_10_-C_16_ alkanes^a^	MYCCH_RS06610	TetR	Coleman et al. (2011a)

a These substrates predicted based on studies of homologous monooxygenases.

### 7.1 Alkane Monooxygenase Regulation: smoXYB1C1Z, hmoCAB, smoABCD

The alkane monooxygenases in *M. chubuense* NBB4 are a curious and diverse group. The *smoXYB1C1Z* gene cluster encodes a group 3 SDIMO that is active on C2-C4 alkanes and alkenes (Martin et al., 2014). There are two putative regulator genes near the *smo* genes; one of these is predicted to encode an AcoR-like regulatory protein that is distantly related to the regulators of other group 3 SDIMOs (BmoR and MmoR; 10% amino acid identity) (Coleman et al., 2011a) and has 30% identity to the closest characterised protein (acetoin dehydrogenase activator AcoR from *B.subtilis* 168 (Ali et al., 2001). The other regulator near the *smo* genes encodes a SigC-like sigma factor; there is no parallel for this in the other group 3 SDIMOs. There is thus far no experimental evidence to confirm that either of these regulatory proteins control *smoXYB1C1Z* expression, but their positioning relative to the *smo* genes indicates that this is likely.

The *hmoCAB* genes encoding a CuMMO are located immediately upstream of the *smoXYB1C1Z* genes in the NBB4 genome (Coleman et al., 2012). This organisation and the common substrates shared by both these monooxygenases (gaseous alkanes and alkenes) suggests that they may be co-regulated. Previous hypotheses about the relationship between the Smo and Hmo enzymes include the possibility that they might be a high- and low-affinity pair or that they are expressed in environments with different cofactor metal availabilities (Martin et al., 2014). The *hmoCAB* cluster is preceded by another *acoR*-like regulator gene, which suggests that the two clusters may have their own independent regulatory systems. It is possible that there are multiple layers of regulation over these two monooxygenases; this would be consistent with a hypothesis that their expression is controlled by both substrate and cofactor availability.

The s*moABCD* cluster of NBB4 encodes a group 6 SDIMO which is thus far uncharacterised except for bioinformatics (Coleman et al., 2011a). The *smoABCD* genes are adjacent to a gene encoding a LuxR-type regulator (van Kessel et al., 2013), which is oriented “head-to-head” with a gene encoding a sensor kinase, with a 29 bp overlap between the 3′ ends of the two genes. It is common for LuxR regulators to belong to two-component transduction systems with a sensor kinase, so it is plausible that this pair of proteins act together in this way. The closest homolog of this NBB4 regulator with a known function is the cold-shock regulator DesR in *B.subtilis* 168, at 26% amino acid identity (Cybulski et al., 2004).

Analysis of the regulation of the *smoABCD* monooxygenase in *Rhodococcus* sp. BCP1 gives clues to the regulation of *smoABCD* in NBB4; these two SDIMOs are very similar, sharing 85–94% amino acid identity in the enzyme subunits. In strain BCP1, the promoter region contains the same core inverted repeat, potential −35 site, and putative catabolite repressor protein binding sites as seen in NBB4 (Cappelletti et al., 2015), along with *luxR* and sensor kinase genes that are 90 and 86% identical, respectively. Based on RT-PCR experiments (Cappelletti et al., 2015) the BCP1 *smo* genes are known to be inducible by C1-C7 alkanes and repressible by glucose or complex media (LB), suggesting the NBB4 *smo* genes are also under similar controls.

### 7.2 Alkane Monooxygenase Regulation: CYP153 and alkB

The CYP153 gene cluster in NBB4 is flanked by genes encoding two regulator proteins—a TetR-like protein and an AraC-like protein (Table 5). The *araC*-like gene and the CYP cluster are divergently transcribed, with just 115 bp separating them; this organisation is consistent with the AraC homolog being the regulator responsible for controlling CYP153 in NBB4, and with promoters of these two genes being located in this 115 bp region. A TetR-like protein is encoded by a gene adjacent to the AlkB-like monooxygenase gene cluster in NBB4. This protein is 31 bp downstream of the *alkB* and putative *rub* genes, and oriented in the same direction as both *alkB* and *rub* genes.

There are many similarities in the configuration of CYP153 and *alkB* in NBB4 and in *Dietzia* sp. strain DQ12-45-1b. The *alkB* homolog in *Dietzia* (*alkW1*) is under the control of a TetR-family repressor; this kind of regulation of *alkB* appears to be unique to the Actinobacteria (Liang et al., 2016b). The regulator *alkX* is 19 bp downstream of the rubredoxin gene in *Dietzia* compared to 31 bp in NBB4. The intergenic DNA region upstream of *alkB* in both species is 53% identical. The −35 and −10 sites of the *alkW1* promoter each differ by just one base from the equivalent *alkB* promoter in NBB4, and the operator sequences in NBB4 have strong homology and similar spacing to those seen in *Dietzia* (Liang et al., 2016a). The configuration of the *araC* family gene in the CYP153 cluster of *Dietzia* is also very similar to the situation in NBB4, i.e., oriented divergently and separated from the ferredoxin gene by 118 bp (Liang et al., 2016b).

If the functional parallels between these monooxygenases in NBB4 and *Dietzia* reflect the sequence similarities, one could infer that the CYP153 and *alkB* genes in NBB4 work as part of a team to efficiently tackle alkanes across a wide range of chain lengths. Nie et al. described how the inducer and substrate range of CYP153 and alkB in *Dietzia* are complementary, i.e., CYP153 hydroxylates *n-*alkanes < C10, while AlkW1 acts on > C10 substrates (Nie et al., 2014b). It is worth noting that NBB4 has two further CYP153 genes (not discussed in detail here), which adds further complications to understanding the relationship between enzymes, inducers and substrates (Coleman et al., 2011a), and highlights the fact that much further work is needed to understand the regulation of alkB and CYP153 in NBB4 and similar bacteria.

### 7.3 Alkene Monooxygenase Regulation: pmoABCD, etnABCD

The substrate ranges of the NBB4 ethene and propene monooxygenases (EtnABCD and PmoABCD, respectively) are similar; both enzymes show activity on C_2_-C_8_ alkenes, with stronger activity on gaseous alkenes (C_2_-C_4_) (McCarl et al., 2018). The fact that strain NBB4 possesses two distinct SDIMO enzyme systems with very close overlap in substrate ranges is unusual and poses questions about why this genotype has evolved and how these genes are regulated. This is an excellent example of why monooxygenase regulatory systems warrant further investigation.

The *pmoABCD* gene cluster in *Mycolicibacterium* NBB4 has a CdaR family regulator encoded by a gene immediately upstream of the monooxygenase, and divergently oriented from these. This family of regulators was originally studied for their role in sugar diacid regulation in *E. coli*, and are typically activators containing a helix-turn-helix domain at the C-terminus (Monterrubio et al., 2000). A similar gene organisation is seen for *pmoABCD* of *Mycobacterium* M156 (Coleman et al., 2011a), although neither regulatory system has been characterised beyond DNA sequencing and bioinformatic analysis.

Regulation of *etnABCD* is likely to be done by a pair of regulators EtnR1/EtnR2, encoded by genes upstream of *etnABCD*. The EtnR1/EtnR2 pair display the typical features of a bacterial two-component regulatory system in which a DNA-binding protein (transcriptional regulator) is phosphorylated by a histidine protein kinase (sensor protein). EtnR1 is a CdaR family protein that contains a DNA-binding helix-turn-helix domain. EtnR2 is distantly related to the DcmR dichloromethane-sensing regulator from *Methylobacterium* DM4 (16% aa identity, 27% aa similarity), and contains a MEDS domain, which is involved in sensing hydrocarbon derivatives in both methanogens and methylotrophs (Anantharaman and Aravind, 2005).

Preliminary research has confirmed an interaction between EtnR1 and a DNA segment containing *etnP*, its putative cognate promoter, *via* EMSA (Moratti et al., 2016). Transcriptomics data (unpublished) also shows a significant upregulation (approx. 8-fold) of both *etnR1* and *etnR2* after exposure of NBB4 cells to ethene; this is consistent with these genes being positive regulators that activate their own expression in a feedback loop, similar to the situation discussed above with AlkS in *P. putida* GPo1. Importantly, the transcriptomics data from NBB4 shows no reads from the short intergenic region thought to contain the *etnP* promoter sequence, consistent with this region driving expression but not itself being expressed.

The *etnR1* and *etnR2* regulators are highly conserved (73–78% aa identity) across many ethene-oxidising isolates, including *Mycolicibacterium* JS623 (e*tnR1*: WP_015305844, *etnR2*: WP_015305843), *Mycolicibacterium tusciae* JS617 (e*tnR1*: WP_006247394, *etnR2*: WP_006247393), and *Mycolicibacterium rhodesiae* JS60 (e*tnR1*: WP_014211282, *etnR2*: WP_014211281). More divergent homologs of these genes (44–59% aa identity) can also be found in many other Actinobacteria, including but not limited to *Streptomyces thermoautotrophicus* H1 (e*tnR1*: WP_066887198, *etnR2*: WP_079045917) and *Amycolatopsis* SYSUP0005 (e*tnR1*: WP_101434350, *etnR2*: WP_158242445). Nearly all these bacteria also contain *etnABCD* homologs near the regulator genes, although most have not been tested for alkene oxidation. Unlike the regulation of *alkB* described above, this suggests that there is overall consistency in the regulation of alkene oxidation across all gram-positive bacteria.

## 8 Constructing a Hydrocarbon Biosensor: General Considerations

There are two general approaches for constructing whole-cell transcription-factor based biosensors. The first approach involves the assembly and expression of the regulatory gene(s), promoters and reporter gene in a plasmid in an appropriate heterologous host strain; this approach allows for more control over expression of the different elements because promoters, ribosome binding sites and other features can be easily and individually modified. The second approach involves the integration of the reporter gene into the genome of the native host, either immediately upstream of the metabolic genes or replacing these; in this situation, there is a gain in stability of the system but the trade-off is that there is less flexibility and construction is more difficult. There are advantages and disadvantages of both approaches, depending on the intended applications of the system (Carpenteret al., 2018).

For environmental applications e.g. in monitoring bioremediation, precise detection at a single cell level is not necessary, and detecting average hydrocarbon concentrations in a population of biosensor cells is acceptable; note that given current legal and biosafety considerations it is more likely that these analyses would be done *in vitro* rather than *in situ*. In a metabolic engineering context, detection at a single-cell level may be desirable e.g. for screening clones in a directed evolution library (Dietrich et al., 2012), and in such applications, a resistance gene may be used instead of a reporter gene to select for the target phenotype (Dietrich, 2011; Carpenter et al., 2018). Challenges to using biosensors at a single-cell level include variations in expression level in individual cells, the potential impact on cell viability, and the impact of the surrounding population on single cells (Tecon and van der Meer, 2006; Carpenter et al., 2018).

The type of replication origin and the host species are key considerations for plasmid-based biosensor construction. Different origins of replication will give different copy numbers, which will impact the signal strength produced by the biosensor (Carpenter et al., 2018). Plasmid-based systems enable the use of hosts that are non-pathogenic, fast to grow and easy to transform, but possible disadvantages include a lack of robustness outside the laboratory, problems with the inducible promoter not being recognised correctly, and potential codon usage issues (Carpenter et al., 2018). Codon optimisation or harmonisation methods can be applied to the regulatory protein and/or to the reporter gene to overcome codon usage problems.

Chromosomally-integrated biosensors will be single-copy systems, and the output signal strength thus correspondingly lower than for plasmid-based systems. Chromosomally-integrated biosensors in non-standard hosts can be more useful for environmental applications, or under harsher physicochemical regimes where traditional host strains would not survive (Jiang et al., 2020). Chromosomal biosensors do not require any selection pressure to maintain, which offers a practical advantage in environmental applications (de las Heras and de Lorenzo, 2010). A major disadvantage of chromosomally-integrated biosensors is the difficulty of construction, since non-standard microbial species have more limited genetic tools and methods available. Homologous recombination has typically been used to make the precise insertions required, but more modern methods such as CRISPR will increasingly replace this (Arroyo-Olarte et al., 2021).

Several problems with chromosomal biosensors relate to interference of different kinds; this may arise from other inducers, other enzyme systems, other global regulators, or other cells in the environment (Su et al., 2011; Jiang et al., 2020). Wild-type microbes that have been engineered to contain a hydrocarbon biosensor circuit may might have other enzyme systems that can metabolise the inducer; this will lead to a depressed response or false negative signals. Less well-studied hosts may contain global control networks that interact with the promoter of interest in unknown ways. For environmental applications or mixed culture conditions, major unknown factors arise from the possible interactions between the biosensor and other microbes; these may create or consume inducers, or enhance/inhibit growth or metabolism in other ways.

## 9 Existing Aliphatic Alkane Biosensor Prototypes

There have been eleven unique hydrocarbon biosensors developed to date (Table 6) using components from four alkane-degrading bacteria (*P. putida, A. borkumensis*, *A. baylyi*, and *T. butanivorans*). These have been designed either for monitoring the bioremediation of oil spills (detection of extracellular alkanes) or to increase our understanding of hydrocarbon metabolism and/or engineer strains for hydrocarbon synthesis (detection of intracellular alkanes, alcohols or aldehydes). The *bmoR*/P_BMO_ biosensor derived from *T. butanivoras* is notable in this collection because it is the only example to date of a SDIMO-associated regulator employed as a biosensor (Dietrich et al., 2012), and because it provides an important reminder that these systems are in some cases induced by metabolites (in this case 1-butanol) rather than alkanes; this is not necessarily a disadvantage, and reflects the broader potential of monooxygenase regulatory systems in biotechnology.

**TABLE 6:** Compilation of existing hydrocarbon biosensors.

Sensing components	Reporter	Host	Type	Inducers and detection limits	Best inducer	Intended application	References
*alkS*/P_alkB_ from *P. putida* GPo1	*luxAB*	*E. coli* DH5α	Plasmid	C_6_-C_10_ linear alkanes, 4–100 nM	C_8_ alkane	Monitoring bioremediation	Sticher et al. (1997)
	*gfp*	*E. coli* DH5α	Plasmid	C_8_ alkane, 10 nM to 1 μM	C_8_ alkane	Monitoring bioremediation	Jaspers et al. (2001)
	*gfp*	*A. borkumensis*	Plasmid	C_8_ alkane, petrol	N/A	Monitoring bioremediation	Sevilla et al. (2015)
	*gfp*	*E. coli* DH10β	Plasmid	C_5_-C_12_ alkanes	N/A	Monitoring bioremediation	Reed (2012)
	*sf-gfp*	*E. coli* DH5αZ1 and *E. coli* HB101	Plasmid	C_8_, C_10_, C_11_ alkanes	N/A	Intracellular alkane detection; characterisation of AlkL	Grant et al. (2014)
*alkJ*/*alkBFG* from *P. putida* GPo1	*luxAB*	*E. coli* TOP10	Plasmid	C_5_-C_12_ alkanes, alcohols, aldehydes 10-200 μM	C_8_ alkane	Intracellular alcohol and aldehyde detection in metabolic engineering	Minak-Bernero et al. (2004)
*alkS*_*AB*_/P_alkB1_ from *A. borkumensis*	*luxAB* or *gfp*	*E. coli* DH5α	Plasmid	C_14_ alkanes, crude oil, 5 nM	C_8_ alkane	Monitoring bioremediation	Kumari et al. (2011)
*alkR*/P_alkM_ from *A. baylyi* ADP1	*luxAB*	*A. baylyi ADPWH_alk*	Chromosomal	C_7_-C_36,_ alkanes and alkenes, 100 μM	C_8_ alkane	Monitoring bioremediation	Zhang et al. (2011), Zhang et al. (2012a), Li et al. (2013)
	*luxAB* and *gfp*	*A. baylyi ADP1*	Chromosomal	C_12_-C_18_ alkanes and aldehydes	C_12_ alkane	Intracellular alkane detection in metabolic engineering	Santala et al. (2012), Lehtinen et al. (2017)
	*gfp*	*E. coli* BL21 DE3 Δ*fadE*	Plasmid	C_15_, C_17_ alkanes	N/A	Intracellular alkane detection in metabolic engineering	Wu et al. (2015)
*bmoR*/P_BMO_ from *T. butanivorans* sp. nov	*tetA-gfp* fusion	*E. coli* DH1 Δ*adhE*	Plasmid	C_3-_C_4_ alcohols, 0.01–100 mM	C_4_ alcohol	Intracellular alcohol detection in metabolic engineering	Dietrich et al. (2012)
				C_4_ aldehyde 1.0–7.5 mM			

### 9.1 Plasmid-Based Alkane Biosensors

More plasmid-based alkane biosensors have been developed than chromosomally-integrated ones, and the majority of these are based on AlkS/P_alkB_ from *P.putida* GPo1 (Table 6). The output signals from these include GFP or LuxAB (detection), or TetA (selection) (Dietrich et al., 2012). Some have the input and output components on separate plasmids (Sticher et al., 1997; Reed et al., 2012), while others use single-plasmid systems (Jaspers et al., 2001; Minak-Bernero et al., 2004; Kumari et al., 2011; Sevilla et al., 2015) (See Figure 1 of Reed et al. (2012) and Figure 1 of Jaspers et al. (2001) for representative plasmid-based biosensor schematics). One novel biosensor uses the monooxygenase genes *alkJ/alkBFG* to convert alkanes to aldehydes, which then support luciferase activity (Minak-Bernero et al., 2004). Another example of note uses AlkR/P_alkM_ from *A. baylyi* ADP1 to measure pentadecane and heptadecane biosynthesis from the *ado* and *aar* genes of *Synechococcus* integrated into an *E. coli* host, *via* a GFP output (Wu et al., 2015). Most plasmid-based biosensors are maintained in lab strains of *E. coli* such as DH5α (Sticher et al., 1997; Jaspers et al., 2001; Kumari et al., 2011), DH10β (Reed et al., 2012) or TOP10 (Minak-Bernero et al., 2004). An exception is one system using *A. borkumensis* as a host; this had a slower response time, but higher sensitivity (Sevilla et al., 2015).

### 9.2 Chromosomal Biosensors

Only two chromosomally-integrated alkane biosensors have been developed to date, both in *A. baylyi* ADP. The first of these (named ADPWH_alk) has *luxCDABE* integrated upstream of *alkM1*, controlled by AlkR/*P*_*alkM1*_ (Zhang et al., 2012b), and has been used for biosensing in an oil-contaminated sample (Li et al., 2013) and further engineered for increased functionality by immobilisation on magnetic nanoparticles (Zhang et al., 2011) (see Figure 1A in Zhang et al., 2012a) for a representative chromosomal biosensor schematic). The other chromosomally-integrated biosensor allows simultaneous detection of intracellular alkanes and aldehydes. In this sensor, a cassette containing a P_alkM-_ *gfp* fusion generates a fluorescent output for alkane detection while a *luxAB* cassette reports on aldehyde concentrations (long-chain aldehydes are the substrate for the luciferase). The system allows reporting on both alkane oxidation to aldehydes and alkane synthesis from aldehydes, since it also contains IPTG-inducible alkane biosynthesis genes (*aar* and *ado*) (Lehtinen et al., 2017).

### 9.3 Benefits and Limitations

The low bioavailability of hydrocarbons is a major challenge for alkane biosensors, which all under-report hydrocarbon concentration by around 20% (Sticher et al., 1997; Kumari et al., 2011; Zhang et al., 2012a; Li et al., 2013). Kumari et al. (2011) found that a longer incubation time was required to increase the bioavailability, and hence detection, of longer chain alkanes (>C_11_), while Sticher et al. (1997) attributed the underestimation to the presence of unknown inhibitor compounds. Li et al. (2013) argued that, despite these shortcomings, the benefits of biosensors still made them valuable, and in the case of their system, the short detection time (0.5–4 vs. 48 h for GC/MS) and the small sample size (1 ml vs. 500 ml required for GC/MS) were major advantages. Biosensors can be surprisingly robust; e.g., Zhang et al. did not observe any instability or loss of function in their ADPWH_alk biosensor, which still worked well after storage for a month in water at 4°C (Zhang et al., 2012a).

## 10 Development and Optimisation of Hydrocarbon Biosensors

An increased understanding of regulatory components opens the door to protein and DNA engineering to optimise the specificity, sensitivity, dynamic range, detection range and response time of these systems for use as biosensors (Ding et al., 2021). The flexibility of regulatory proteins in detecting multiple inducers can be seen as a double-edged sword (Diplock et al., 2010); this may be useful in nature for a bacterium to respond to multiple possible carbon sources, but may not be ideal for biosensing of specific analytes. Factors targeted for optimisation include host strain, promoter sequence, replication origin, ribosome binding site, protein-promoter binding sites, and the sequence of the regulatory protein; the latter may also involve addition of degradation tags to reduce the metabolic burden on the cell (Ding et al., 2021).

Techniques such as site-directed mutagenesis, random PCR mutagenesis and DNA shuffling can be used to generate libraries of regulator variants with improved functions (van der Meer and Belkin, 2010). Regulatory proteins of the AraC/XylS and TetR families have successfully been mutated to alter binding specificity (Galvão et al., 2006); e.g., the R41G mutation in XylS increases the response to 2-ethylbenzoate, while reducing the response to 2-methylbenzoate (Galvão et al., 2006). The crystal structure of a protein can reveal optimal sites for mutation; this approach was successfully used in the case of DntR, a salicylate-induced activator in *P. putida*, to identify the binding pocket (Lönneborg et al., 2007).

An impressive example of optimisation involved the directed evolution of *AlkS* in an *E. coli*-based *AlkS/P*_*alkB*_ biosensor (Reed et al., 2012). Two rounds of error-prone PCR resulted in the identification of several mutations that conferred improved response to short-chain alkanes (C_5_-C_9_), with the best mutant showing a five-fold increase in fluorescence output in response to hexane compared to wild type AlkS. The Q410K and S470T mutations were present in the two best mutants; the latter increases the bulkiness in the putative alkane binding pocket, discouraging binding of larger alkanes (Reed et al., 2012). This study provides clear evidence for the usefulness of directed evolution approaches to alter the substrate range and specificity of alkane-sensing systems.

A novel approach to biosensor development was taken by Zhang et al. (2011), who functionalised the *A.baylyi* ADPWH_alk biosensor using magnetic nanoparticles, to allow for remote manipulation of the reporter cells; this allows biosensor cells to be collected for re-use after deployment in a complex environment by application of a magnetic field. The magnetic nanoparticles had no adverse impact on cellular function or alkane detection and could be attached with an efficiency of 99.96%. The ADPWH_alk biosensor has a shorter response time (30 min) compared to a previously-developed biosensor using the same AlkR*/P*_*alkM*_ components (10 h) (Ratajczak et al., 1998a); this is potentially attributable to three fortuitous point mutations located near the AlkR binding site (Zhang et al., 2012b).

The 1-butanol biosensor constructed using BmoR/P_BMO_ and GFP has also been optimised (Dietrich, 2011). In this case, lower temperature (25°C) and lower levels of BmoR expression resulted in a more robust biosensor. A synthetic ribosome binding site for *gfp* expression was also beneficial, resulting in higher fold-induction and better dynamic range. Other parameters that were optimised included: induction time (early exponential phase was best), inducer concentration (alcohol toxicity was observed above 40 mM 1-butanol), host strain (Δ*adhE* strain lowered background fluorescence) and plasmid origin of replication (a low copy replicon gave no fluorescence).

Calibration of biosensors to traditional detection techniques is a crucial step towards real-world application, as it proves the integrity and reliability of the device. This was done with the *A. baylyi* ADPWH_alk biosensor, by comparing it to GC/MS measurements (Li et al., 2013). Two contaminated soil samples (>5,000 mg petroleum/kg soil) were analysed along with two clean soil samples from an adjacent site. It was found that the biosensor reported ∼20% lower levels of oil compared to the GC/MS, which could be attributed to the low bioavailability of alkanes, as discussed above (Sticher et al., 1997). Inducer bioavailability has been a consistent problem for biosensor development, but a counter-argument can also be made that the bioavailable fraction is more relevant than the total hydrocarbons for determining ecotoxicity (Tecon and Van der Meer., 2008). Enhancing the uptake of alkanes may increase their apparent bioavailability; e.g., co-expression of the AlkL transporter in an *E. coli* strain expressing the AlkB monooxygenase resulted in a 100-fold increase in oxidation of large alkanes (>C_12_) (Grant et al., 2014).

A recent biosensor optimisation study examined the impact of different host organisms on the performance of the biosensor. A dual plasmid biosensor containing AlkS and *P*_*alkB*_ with a GFP reporter was transformed into several alkane-assimilating marine bacteria, and also into *E. coli* (Sevilla et al., 2015). Although *E. coli* had the fastest detection rate, *A. borkumensis* was the best candidate overall, giving the most sensitive detection of octane (detection limit of 0.5 µM), and also effectively detecting C_7_-C_9_ alkanes at 0.012% v/v concentration in a saltwater sample. This is an excellent example of how a hydrocarbon biosensor can be optimised for a specific application—in this case, monitoring bioremediation of oil spills in oceans—by changing the host organism of the sensor.

## 11 Bringing Hydrocarbon Biosensors to Market

Despite the above-described successes with optimisation, no hydrocarbon biosensors are commercially available at the time of writing. The deployment phase of a whole-cell microbial biosensor is challenging, particularly for devices that have environmental applications. The use of microbial biosensors in the environment and the associated challenges with their deployment have been extensively reviewed (D’Souza, 2001; van der Meer et al., 2004; Harms et al., 2006; de las Heras and de Lorenzo, 2010; Coleman et al., 2011a; de las Heras and de Lorenzo., 2012; Plotnikova et al., 2016; Shemer and Belkin, 2019; Hicks et al., 2020; Jiang et al., 2020); here we will focus on a few key engineering considerations relevant to the specific case of hydrocarbon biosensors. The potential of these systems has been long acknowledged, and yet the same barriers seem to stand in the way, decades after they were first recognized. These barriers include legal considerations surrounding the release of genetically manipulated bacteria, financial issues about the investment of time and resources required to yield sufficiently optimised systems for field application (Sadana and Sadana, 2011), and functional limitations of these biosensors, such as bioavailability concerns (van der Meer, 2016).

Two linked challenges for biosensor commercialisation are culture scale-up and immobilisation. Large numbers of cells need to be grown in pure culture and kept viable and at high activity; continuous culture is a good option here, but this requires more complex equipment and maintenance and has a higher risk of contamination (Bjerketorp et al., 2006). Cells then need to be preserved, immobilised and/or contained such that they can be deployed safely and effectively without compromising their functionality; these methods may include freeze- or vacuum-drying, immobilisation and/or encapsulation. Cells can be immobilised on solid surfaces like optical fibres or microchips, or encapsulated in soft materials like hydrogels, sol-gel, carrageenan, alginate, polyacrylamide, oxysilane or polyvinyl alcohol (Liu et al., 2007). The choice of encapsulation/immobilisation methods impacts many aspects of biosensor function, such as the rates of gas and solute diffusion, biosensor response time, cell viability, and shelf-life.

Striking a balance between functionality and biosafety is an ongoing challenge for all whole cell-based biosensors that have intended applications outside of the laboratory. The inclusion of toxin/anti-toxin systems, non-canonical amino acids, kill switches, engineered auxotrophy, or conditional origins of replication are examples of methods to prevent unintentional gene transfer from the biosensor to the environment (Wright et al., 2015; Hicks et al., 2020). Given the high cost and labour inputs into their production, the potential to reuse or recycle these biosensors should also be considered (e.g., see above-described example using magnetic nanoparticles (Zhang et al., 2011). If the biosensor is intended for single use, biodegradation options should be assessed, e.g., by choosing a biodegradable immobilisation surface. Testing the function of a biosensor in its intended application environment is very important since other chemical compounds or microbes in the target sample could inhibit or confound its response (van der Meer and Belkin, 2010); elucidating these interactions should be an early focus of the deployment phase of biosensor development.

## 12 Future Research Priorities and Conclusions

One useful analogy for the landscape of aliphatic hydrocarbon biosensor development is the research funnel. Identifying the bottlenecks in this funnel is the key to efficiently directing research for different kinds of biosensors. At the top of the funnel, there is an abundance of putative monooxygenase and other hydrocarbon catabolic genes in databases that have been tentatively identified using bioinformatics. There is then a steep decline at the next level, representing experimentally characterised catabolic systems, with only a few dozen monooxygenases reaching this milestone. The identification and characterisation of the regulatory systems at the next two lower levels filter the candidates even further. Only a handful of biosensors make it to proof-of-concept stage, with optimisation and development attempts made on an even smaller subset of these, and thus far no candidates have been deployed as commercial products.

The large number of putative monooxygenase genes already existing in databases reflects the constantly decreasing cost of DNA sequencing, and the availability and accessibility of bioinformatic software; this part of the funnel is not the best focus for efforts to develop biosensors. The characterisation of monooxygenases is also not a major limiting factor since many representatives of different monooxygenases have now been at least partially characterised. In contrast, there are strong arguments for focusing research efforts on identifying and characterising regulatory genes and promoters. Extrapolating from previous well-studied systems is of limited usefulness, as previous work suggests different species may have unique regulatory mechanisms, even for similar catabolic genes (Moreno and Rojo, 2019). More effort is needed to overcome a bias in the literature towards C_5_-C_18_ alkane sensing systems and AlkB or CYP enzymes; this has led to a neglect of the systems responding to smaller alkanes and alkenes, especially those associated with SDIMOs and CuMMOs. Investing in research on thorough characterisation of regulatory systems will give increased understanding of how these systems function in nature, leading to better biosensors, and also helping to remove legal barriers to implementation in the field.

Very few hydrocarbon regulatory systems have met all five proposed criteria for complete characterisation (van der Meer and Belkin, 2010), and these knowledge gaps will limit the development of biosensors. The complexities of these systems should not be underestimated, and much more research is required to appreciate their intricacies. Untangling the regulation of different hydrocarbon catabolic genes that exist within a single strain is an especially useful avenue to pursue (Coleman et al., 2011a); this will give insights into the behaviour of biosensor circuits which must function in the presence of other hydrocarbon catabolic genes and regulators, and will inform the construction of more complex systems that integrate multiple sensing systems into a single cell.

Continued research attempts at the proof-of-concept stage of hydrocarbon biosensors are also warranted. This may involve directed evolution or site-specific modifications of regulatory components to yield biosensors with improved qualities, or proceeding with wild-type sensor systems, many of which already have good sensitivity and specificity (van der Meer and Belkin, 2010). Challenges at the proof-of-concept stage are often due to issues with robustness, shelf-life, and applicability to different real-world environments (Hicks et al., 2020) e.g., how to safely immobilise the cells while maintaining their function. Investigation into the localisation of wild type regulatory proteins could also be valuable, as it can influence the response time of the biosensor (Ding et al., 2021).

**FIGURE 5:**
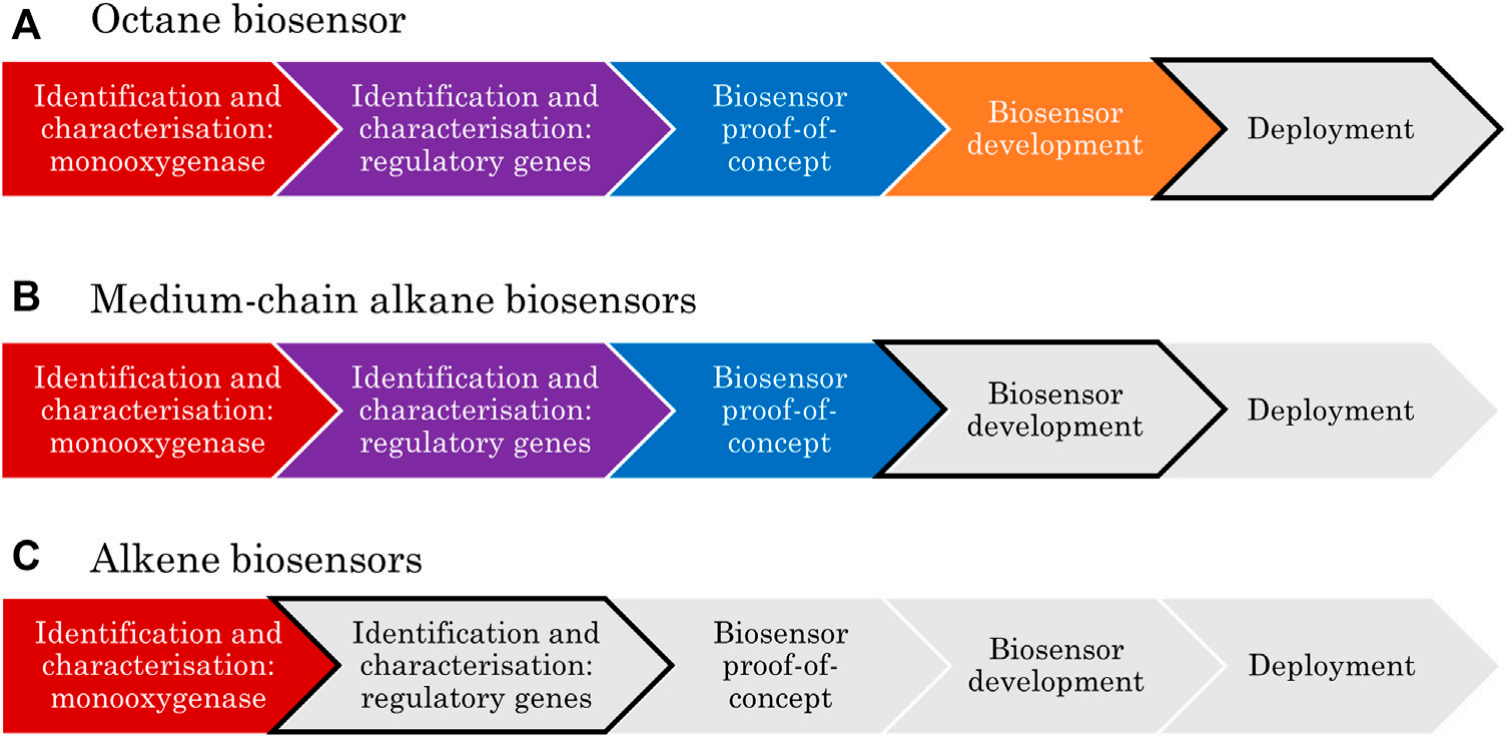
Status of development of octane **(A)**, medium-chain alkane **(B)**, and alkene biosensors **(C)**, with priority research areas yielding maximum impacts indicated by the dark outlined arrows.

Three specific recommendations for research can be drawn from this review (Figure 5). Firstly, there are already two excellent octane biosensors, the plasmid-based *AlkS/P*_*alkB*_/GFP biosensor in *E. coli* DH10β (Reed et al., 2012) and the chromosomal *alkR/P*_*alkM*_ */luxAB* sensor in *A. baylyi* (Zhang et al., 2011; Zhang et al., 2012a; Li et al., 2013); these are ready to progress to the final stage of development and deployment, which should focus on testing their robustness, sensitivity and selectivity in various real-world environments, and on finding the best methods of immobilising or encapsulating the cells. Secondly, several medium-chain alkane biosensor systems that work well in the lab (Zhang et al., 2012b; Sevilla et al., 2015) should now be progressed to the optimisation stages, e.g. to make a suite of sensors, each with selectivity for different single analytes. Finally, the molecular details of alkene-sensing systems need to be much better characterised, since our understanding of these is still rudimentary.

New developments in synthetic biology have greatly expanded the possibilities for hydrocarbon biosensors. The combination of synthetic biology methods with the wealth of novel sequences that continue to appear in genetic databases promises an exciting future for this research field. However, to maximise these possibilities our efforts must be effectively targeted at the appropriate development stages for each biosensor. Making successful commercial biosensors for deployment in real environmental or industrial contexts will require genuinely interdisciplinary efforts including microbiologists, molecular biologists, biochemists, structural biologists, engineers, materials scientists, and mathematical modellers. The microbes have provided the raw materials, but now we must provide the ingenuity and the effort to complete these tasks.
